# Elucidating the role of medicinal plants in melanoma treatment: a review on metastatic progression and phytochemical intervention

**DOI:** 10.3389/fphar.2026.1821643

**Published:** 2026-08-28

**Authors:** Jonathan Lloyd Seaman, Danielle Twilley, Namrita Lall

**Affiliations:** Department of Plant and Soil Sciences, University of Pretoria, Pretoria, South Africa

**Keywords:** anticancer, antimelanoma, medicinal plants, metastatic cascade, natural products, phytochemicals

## Abstract

Metastatic melanoma remains one of the most highly aggressive and treatment-resistant cancers, highlighting the need for enhanced understanding of its progression and novel therapeutic strategies. The metastatic cascade is an intricate, dynamic, and coordinated series of events which initiates with melanoma cell proliferation and the release of regulatory signalling molecules, followed by angiogenesis in response to hypoxia and nutrient deprivation, and extracellular matrix (ECM) degradation and invasion via matrix metalloproteinase (MMP) activity. Intravasation generates circulating tumour cells (CTCs), which evade immune surveillance through platelet shielding before extravasating at distant sites to establish secondary tumours. Conventional metastatic melanoma treatments, including surgery, chemotherapy, radiotherapy, targeted inhibitors and immunotherapy, are associated with significant limitations, which has shifted the focus to the use of interventions obtained from natural product resources. In light of this, this narrative review aims to emphasise the potential of plant-derived candidates with anti-melanoma activity, including medicinal plant extracts and isolated phytochemicals, by bridging the gap between traditional medicine and modern oncology and permitting medicinal plants to serve as a compelling avenue for future melanoma management. Owing to the substantial heterogeneity in experimental models, extraction methods, phytochemical composition, and outcome measures, a quantitative comparison was deemed inappropriate, and the evidence was synthesised narratively. It was determined that several medicinal plants and their constituent phytochemicals demonstrate considerable promise as multitargeted therapeutic candidates against metastatic melanoma. However, the predominance of early-stage preclinical studies focused primarily on proliferation, limited investigation of the later stages of metastasis, insufficiently explored and documented long-term *in vivo* toxicological effects, and the absence of melanoma-specific clinical trials are major limitations to their clinical translation. To translate this potential into evidence-based melanoma treatments, future research should prioritise comprehensive antimetastatic, toxicological, and clinical investigations, and address key considerations such as the multi-target activity of plant extracts, standardisation, the importance of model selection, assumptions on botanical safety, bioavailability, and regulatory compliance.

## 1 Introduction

Malignant melanoma, a form of skin cancer arising from pigment-producing melanocytes, is one of the most aggressive and deadly types of skin cancer (Cummins et al., 2006; DeSanto et al., 2023). A common fallacy in the human perception of disease and illness is that unless a disease is highly prevalent, it must be of minimal significance and concern; however, despite comprising only 1% of skin cancer cases, melanoma is responsible for over 90% of skin cancer-related deaths due to its high potential for metastasis (Eddy and Chen, 2020; Saginala et al., 2021; Siegel et al., 2020). In the United States of America alone, approximately 234,680 cases of melanoma are estimated to be diagnosed in 2026, with a predicted 122,680 non-invasive and 112,000 invasive cases (American Cancer Society, 2026). Concurrently and as a testament to the accelerated development of this disease and its aggressive malignant profile, investigation into melanoma incidence trends has revealed a 320% increase over the period of 1975–2018 (Saginala et al., 2021).

Worldwide, the incidence of melanoma diagnoses has shown a consistent and significant increase, posing a threat to the global disease burden and unearthing a unique challenge to biomedical researchers. Early detection and treatment of melanoma are imperative for improving survival rates and for controlling melanoma epidemiology and prognosis. Although melanoma has been recognised as a notably lethal form of skin cancer, the overall 5-year survival rate has been calculated as approximately 94.7% (SEER, 2026). It must, however, be noted that this 5-year rate is unique in how patients present with the disease. Localised melanoma or non-metastatic melanoma accounts for 78% of melanoma cases upon initial patient presentation at a healthcare facility. In first-time consultations, less than 5% of cases involve the diagnosis of late-stage metastatic disease (Plaza et al., 2010; Waseh and Lee, 2023). Localised melanoma, classified as Stage I, if accurately and effectively managed, may pose only minor cause for concern. Advanced Stage III and IV disease are associated with 5-year survival rates incomparable to those associated with localised forms of the disease. Following haematogenous or lymphatic dissemination, melanoma acquires the definitive classification of being metastatic, a classification with poor prognosis, progressive disease deterioration and a 5-year survival rate of between 5% and 19% (Sandru, 2014).

Metastatic progression is a multifaceted cascade intricately linked to the aggressiveness of melanoma, its dissemination throughout the body, and the difficulty of therapeutic management of this disease. The process is initiated upon the proliferation of primary melanoma cells, cellular migration through tissue via the use of migratory factors and associated ECM invasion and destruction (Lan et al., 2024). Vascular access is one of the primary and essential disseminative routes for metastatic cells, and thus their adhesion to endothelial cells lining the vasculature and their subsequent trans-endothelial migration (intravasation and extravasation) promotes the ability of metastatic cells to migrate to distant sites and form micro-metastatic colonies existing in unique physiological niches (Van Zijl et al., 2011). Aberrant blood vessel formation through accelerated angiogenesis, coupled with drainage into lymphatic systems and migration via perineural networks, ensures that melanoma has numerous potential mechanisms by which it may ensure maximum colonisation (Fares et al., 2020; Lee et al., 2019; Van Zijl et al., 2011). Although metastatic melanoma may form distant populations of cells at any location throughout the human body, metastatic populations have a particular preference towards certain cell types and organs such as the lung, brain, liver and bone (Damsky et al., 2014). The acquisition of an additional type of cancer at a secondary site introduces its own category of complications and tumour burden, impacting not only the affected organ but also creating further potential for metastasis and immunological challenges.

Despite significant strides in understanding and treating malignant melanoma, several critical gaps in knowledge regarding therapeutic efficacy and management remain. Currently, there is no pharmacological agent which actively inhibits all stages of the metastatic cascade; however, there are acutely targeted therapies which inhibit a particular gene or the proteinaceous product of that genetic instruction (Maverakis et al., 2015; Switzer et al., 2022). The primary challenge originates when diagnostic methods fail to identify malignancies (particularly non-cutaneous melanomas) at a treatable stage. Moreover, given that melanoma displays a physiologically heterogeneous and molecularly diverse profile, complications have arisen regarding the development of universally effective treatments. While targeted therapies and immunotherapies have shown promise, their success is limited by the development of resistance and adverse side effects and, therefore, the identification of novel therapeutic compounds is critical in the attempt to counteract drug resistance and improve patient outcomes (Dixon et al., 2024). As the world’s awareness of synthetic compounds is dynamically remodelled, with a shift towards a more natural approach, medicinal plants have emerged as a promising source of bioactive molecules for the management of metastatic melanoma, with expanding evidence supporting their therapeutic potential.

Despite the fact that medicinal plant extracts and isolated phytochemicals have demonstrated noteworthy antimelanomagenic and antimetastatic potential, it is becoming increasingly challenging to translate this progress into clinical practice, as these therapies remain constrained by inconsistencies in experimental modelling and safety evaluation. Historically, phytotherapeutic investigations in melanoma have relied heavily on two-dimensional (2D) monolayer cultures for cytotoxic screening and mechanistic exploration (Beaumont et al., 2014). Reconsideration is therefore necessary, as these *in vitro* models oversimplify tumour architecture and neglect the intricacies of the ECM, stromal, and immune components associated with tumour cells. Subsequently, this may overestimate bioactivity due to the ease of penetrating a monolayer (Marconi et al., 2018; Rebecca et al., 2020). More physiologically representative models, including three-dimensional (3D) spheroids and skin reconstruct models, draw attention to cell-cell and cell-matrix interactions, nutrient and oxygen diffusion gradients, as well as tumour heterogeneity - elements typically absent in 2D models (Raza et al., 2017). Nonetheless, advances in evaluating phytotherapeutic formulations have moved toward more controlled and reproducible models that better emulate the complexity of tumours within the sphere of systems biology. As an alternative, xenograft platforms enable evaluation of tumour growth kinetics and metastatic dissemination; however, the reliance on immunocompromised hosts limits the evaluation of botanical products with potential immunomodulatory activity (Ny et al., 2020). To circumvent this issue, researchers have shifted to immunocompetent systems, such as syngeneic models and genetically engineered mouse (GEM) models. These models investigate immune-mediated tumour control within native tissue environments and are directly aligned with the profound emergence and surge in immune checkpoint inhibitor studies (Carlino et al., 2021). GEM models facilitate the generation of an experimental environment that closely mimics human melanoma, thereby allowing researchers to more clearly study tumour characteristics, progression, resistance mechanisms, metastasis and responses to therapies within a fully functional immune environment (Kersten et al., 2017). Overall, each evaluation model suggests a balance between strengths and weaknesses, emphasising the necessity of integrative multi-platform strategies, even though developing plant-derived products for melanoma requires more than merely demonstrating bioactivity *in vitro* or *in vivo*. Beyond the preliminary and mechanistic data, practical considerations such as extract standardisation, complex dosing strategies, poor bioavailability, restrictive regulatory frameworks, and multi-target effects must be addressed to ensure favourable and consistent clinical outcomes. Finally, comprehensive clinical trials, as well as toxicological evaluations using acute, sub-chronic, chronic and organ-specific toxicity modalities, are limited in species displaying noteworthy antimelanoma activity. The only way to elevate exploratory investigation of plant-derived products as a viable adjunctive therapeutic intervention against metastatic melanoma is to recognise the importance of these prerequisites for clinical translation and to incorporate safety profiling alongside robust bioactivity data. In support of this, it is essential to note that medicinal plants are often perceived as intrinsically safe due to centuries-old use; however, natural origin does not equate to safety. Many phytochemicals possess potent biological activity that may confer both therapeutic efficacy and dose-dependent toxicity, which emphasises the need for rigorous preclinical and clinical safety evaluations to establish their therapeutic and toxicity window.

This review seeks to address the use of medicinal plants as biologically rich sources of antimetastatic activity and structurally diverse bioactive compounds, and as hosts with the potential to modulate multiple molecular pathways involved in tumour progression, metastasis, and immune evasion, thereby offering advantages over single-target synthetic agents. In doing so, it also considers the practical hurdles in translating these compounds into clinically relevant interventions, including the choice of experimental model, dosing, bioavailability, extract standardisation, safety evaluation, and regulatory compliance.

## 2 Methodology

This narrative review was conducted to provide a comprehensive reflection of the current evidence base regarding the potential role of medicinal plants in the management of metastatic melanoma and was not completed as a formal systematic or scoping review. A structured literature search was conducted (01/02/2025–03/07/2026) using the electronic databases PubMed, Scopus, Web of Science, ScienceDirect and Google Scholar to identify relevant literature published between 1970 and July 2026. This period was selected in an attempt to capture both the historical fundamentals of melanoma biology and medicinal plant research, whilst concurrently addressing and incorporating recent advances in phytochemistry and targeted cancer therapeutics. Additional studies were identified through manual screening of reference lists taken from the initial eligible publications selected for this study. In addition, citations to key articles were identified, and, where appropriate, relevant books, book chapters, conference proceedings, patents, and authoritative reports were included. Search terms included, but were not limited to, “melanoma,” “metastatic melanoma,” “metastatic cascade,” “melanoma growth factors,” “anti-proliferative,” “angiogenesis,” “invasion,” “intravasation,” “extravasation,” “immune evasion,” “tumour microenvironment,” “medicinal plants,” “phytotherapy,” “phytochemicals,” “natural products,” “anticancer plant extracts,” “antimetastatic phytochemicals,” “drug-herb interactions,” “combination therapy,” “melanoma models” and “toxicity.” In an attempt to maximise the sensitivity and specificity of the search, Boolean operators (AND/OR) and database-specific search filters were applied. As a means of minimising selection bias, the literature presented in this review was selected on the basis of scientific relevance, methodological quality, and contribution to the review’s objectives, rather than a focus on study outcomes or reported efficacy. When eligibility was considered, inclusion criteria included studies reporting plants with anti-melanoma activity, inhibition of metastatic processes, immunomodulatory mechanisms, phytochemical characterisation, toxicity and safety evaluation, pharmacokinetic properties, and interactions with conventional anticancer therapies. Only English-language publications were included. Studies were excluded if the experimental details or controls were inadequate; if they focused on non-melanoma cancers in isolation rather than in a metastatic context; if they duplicated previously published findings; or if they originated from non-peer-reviewed sources without a sufficient degree of scientific validation. Scientific names, taxonomy and reported phytoconstituents were verified using authoritative botanical databases and phytochemical resources. As a result of the profound degree of heterogeneity that exists in experimental melanoma models, extraction procedures, phytochemical composition, dosage regimens, and outcome measures across studies, quantitative comparison or meta-analysis was deemed inappropriate. Instead, the findings of this review were synthesised narratively, with an evaluation of the current state of evidence regarding bioactive potential, biological plausibility, the strength of the experimental model, and overall translational relevance.

## 3 Principles of melanomagenesis and metastatic proliferation

### 3.1 Melanocyte biology and melanoma pathology

Melanocytes are neural crest-derived, dendritic, pigment-producing cells that migrate to the dermis during embryogenesis, where they regulate cutaneous pigmentation through coordinated extracellular and intracellular signalling mechanisms (Cui, 2023; Petit and Larue, 2016; Sulaimon and Kitchell, 2003). Beyond the skin, melanocytes are distributed in the hair follicles, uveal tract of the eye, central nervous system leptomeninges, substantia nigra, cardiac tissue, mucosal surfaces, inner ear and other specialised tissues, thereby explaining the occurrence of both cutaneous and non-cutaneous melanomas (Hutcheson et al., 2021; Ikeda et al., 2021; Kaucka et al., 2021; Plonka et al., 2009; Polisetti et al., 2021; van Beelen et al., 2020). Melanin, a melanocyte pigment, provides inherent protection from ultraviolet (UV) radiation-induced damage. Skin phototype, or the degree of skin pigmentation, has been identified as an important predictive tool in skin cancer risk analysis, although delayed diagnosis in darker phototypes may contribute to poorer clinical outcomes (Gupta et al., 2016; Lin and Fisher, 2007). In addition to pigment production, melanocytes contribute to innate immune defence via phagocytic activity, cytokine secretion, and antigen presentation (Gasque and Jaffar-Bandjee, 2015; Le Poole et al., 1993).

Disruption of melanocyte homeostasis by environmental and/or genetic instability can lead to melanoma development, an aggressive neoplasm arising from clonal expansion of melanocytes and malignant transformation. Melanomas are classified into one of two broader and more general categories, those being cutaneous (approximately 90% of cases) and non-cutaneous melanoma (approximately 10% of cases), the latter being commonly associated with aggressive clinical behaviour and late-stage presentation (Bennett et al., 1994; Eddy and Chen, 2020; Kuk et al., 2016; Wilkins and Nathan, 2009). Ultimately, melanoma susceptibility is determined by the intricate interactions between genetic predispositions and biological or environmental perturbations. Despite the inherent complexity of melanoma pathophysiology, it is a disease that, with active avoidance of risk factors, can be managed more effectively.

### 3.2 Melanoma risk factors

The emergence of skin cancer is influenced by a multitude of risk factors. Individuals with increased solar exposure, numerous or atypical nevi, fair skin, lighter hair pigmentation, and hereditary predisposition are at heightened risk (Gandini et al., 2005b). While environmental exposure and genetic susceptibility are well recognised, melanocyte-specific characteristics, particularly melanogenesis and melanosome synthesis, also contribute significantly to melanomagenesis. There is thus a need for a balanced and integrative approach to melanoma management that accounts for all risk factors.

#### 3.2.1 Environmental and genetic risk factors

Ultraviolet (UV) radiation is a predominant etiological factor in the development of melanoma, with solar radiation being the principal source. Although a discrepancy exists regarding the weight that UV-A or UV-B radiation holds in melanoma progression, recent research has provided reasonable justifications for both, thereby highlighting the importance of both wavelength ranges. It has been determined that UV-A wavelengths contribute to the induction of the Warburg-like effect and substantially alter melanoma cell metabolism. This is achieved by increasing glucose uptake and lactic acid production, thereby upregulating MMPs and urokinase-type plasminogen activator (uPA), promoting melanoma cell invasion and proliferation (Kamenisch et al., 2016). In other experimental studies, it has been suggested that UV-B radiation is more influential than UV-A radiation from a genetic perspective. The UV-B wavelength range of 280–320 nm has been shown to be more than 1,000 times as genotoxic per photon as UV-A (320–400 nm) light, despite daily UV-A exposure exceeding UV-B exposure (Arisi et al., 2018; Marionnet et al., 2015).

Apart from UV radiation, a familial background of melanoma represents a significant genetic contributor to disease susceptibility, particularly when first-degree relatives are affected. It is estimated that close to 10% of melanoma diagnoses arise within the context of families (American Cancer Society, 2026). In terms of inherited predisposition, a distinct set of mutations at specific allelic loci is associated with familial forms of melanoma. In much the same way, there are additional categories of spontaneous mutations that serve as key oncogenic drivers in non-familial contexts. The above-mentioned mutations are found across a diversity of patient profiles, not only in sun-exposed and familial settings but also in UV-independent settings. Table 1 summarises common examples of familial, UV-induced, and UV-independent mutations that play an integral role in melanoma development, along with their associated biological functions and clinical significance.

**TABLE 1:** An overview of mutations commonly associated with melanoma, together with their biological role and clinical significance.

Gene	Melanoma subtype	Biological role	Clinical significance	References
B-Raf proto-oncogene (BRAF)	Cutaneous	Mitogen-activated protein kinase (MAPK) activation	Most common driver mutation	Edlundh-Rose et al. (2006), Saginala et al. (2021)
N-Ras proto-oncogene (NRAS)	Cutaneous	MAPK activation	Early oncogenic driver	
Neurofibromin 1 (NF1)	Chronically sun-induced damage (CSID)	Negative regulator of RAS	Loss activates MAPK signalling	Lu et al. (2025)
KIT proto-oncogene (KIT)	Acral, mucosal	Receptor tyrosine kinase	Therapeutic target in selected patients	Beadling et al. (2008)
G protein subunit alpha Q (GNAQ)	Uveal	G-protein signalling	Common uveal melanoma mutation and UV-independent	Van Raamsdonk et al. (2010), Violanti et al. (2019)
G protein subunit alpha 11 (GNA11)	Uveal	G-protein signalling		
Melanocortin 1 receptor (MC1R)	Germline susceptibility	Pigmentation	Associated with fair skin phenotype	Mitra et al. (2012)
Cyclin-dependent kinase inhibitor 2A (CDKN2A)	Familial melanoma	Cell-cycle regulation	High-risk inherited mutation	Saginala et al. (2021)

In summary, focus on preventive interventions regarding environmental factors such as UV radiation exposure has dominated melanoma risk management; however, while imperative, UV exposure does not fully account for the variability in melanoma risk and outcomes among individuals, which often have a genetic basis. Additionally, to gain a more comprehensive understanding of melanoma pathogenesis, it is essential to explore additional biological risk factors that contribute to susceptibility.

#### 3.2.2 Biological risk factors

As with all other cancers, dermatological malignancies are often deeply affected by immune function. Individuals with compromised immune systems, whether this be due to underlying medical conditions or immunosuppressive therapies, often exhibit a heightened propensity towards malignancy. A relevant example lies in the case of organ transplant recipients who are frequently administered immunosuppressive medications to prevent organ rejection. The challenge is that this inadvertently elevates their melanoma risk as a result of immune system destabilisation. Similarly, individuals infected with the human immunodeficiency virus (HIV), the causative agent of acquired immunodeficiency syndrome (AIDS), characteristically experience immune suppression, which further increases their vulnerability to melanoma (Kubica and Brewer, 2012).

Another noteworthy biological risk factor associated with melanoma initiation and progression is the presence of nevi, also known as moles. Moles are benign, non-cancerous aggregates of melanocytes; however, despite their benign nature, they are considered to serve as both melanoma risk enhancers and melanomagenic precursors (Saginala et al., 2021). Confirmation of their melanomagenic potential was demonstrated in a study in which those with more than 100 mol were found to be at a sevenfold increased risk of developing melanoma relative to those with fewer than 15 mol (Gandini et al., 2005a). Atypical and dysplastic nevi are well-known markers of enhanced melanoma risk, particularly in individuals with dysplastic nevus syndrome or familial atypical multiple mole and melanoma (FAMMM) syndrome. Both of these conditions are associated with a markedly elevated lifetime risk of melanoma (Greene et al., 1987). In addition, the red hair/fair skin phenotype, frequently linked to inactivating polymorphisms of the MC1R gene, constitutes the highest-risk phenotype for melanoma development when pigmentation is considered (Mitra et al., 2012).

Ultimately, the cumulative interaction of genetic susceptibility, biological factors and environmental risk exposure initiates a complex series of events which lead to melanoma progression and an elevation in invasive potential. Although local melanomas are associated with a high curative rate, metastatic advancement via the metastatic cascade leads to fundamentally altered therapeutic outcomes and survival rates. It is thus essential to prevent the convergence of risk factors to limit disease burden and avoid the multifaceted dissemination of melanoma cells to distant sites.

### 3.3 Advanced malignancy and the pathology of metastatic melanoma

**FIGURE 1:**
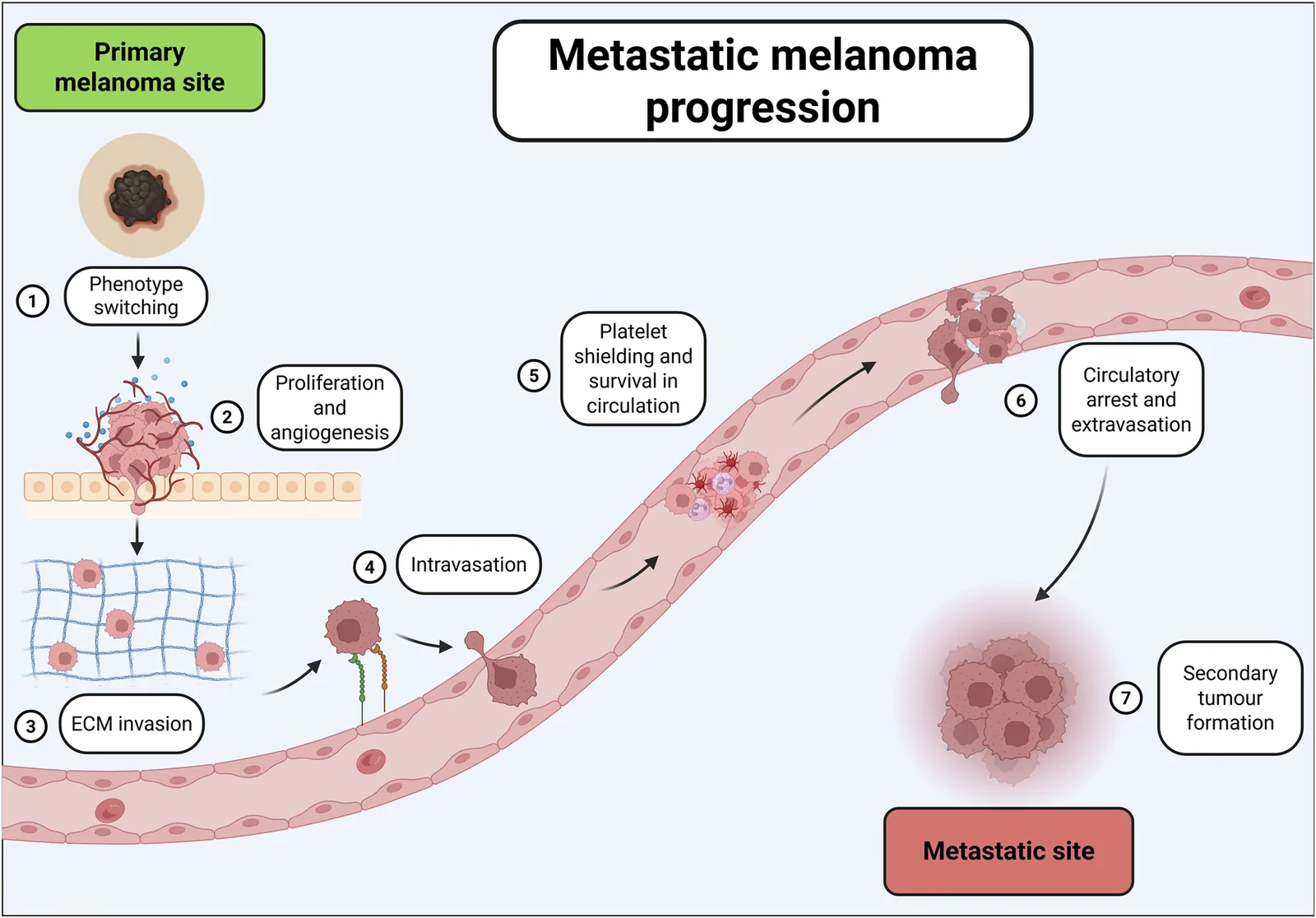
An overview of the metastatic cascade highlighting the critical stages of proliferation, angiogenesis, ECM degradation and invasion, intravasation, survival in circulation, extravasation and finally colonisation (Created in BioRender. Seaman et al. (2026) https://BioRender.com/5ose0p6).

Metastasis is a complex, multi-phase process involving several critical stages. Initially, tumour cells invade surrounding tissues and degrade the extracellular matrix, then attach to and enter blood vessels via a process known as intravasation. Once in circulation, the metastasising cells must survive the hostile environment of the bloodstream, which is plagued by threatening immunomodulatory compounds, including circulating immune cells, cytokines, and complement proteins that can recognise and eliminate foreign or abnormal cells. Should circulatory survival be accomplished, metastasising cells then initiate exit from the blood vessels (extravasation) and establish themselves in distant organs, where they proliferate and form secondary tumours (Eger and Mikulits, 2005; Massagué and Obenauf, 2016; Valastyan and Weinberg, 2011). A summative overview of this process is provided in Figure 1.

Despite this elaborate sequence, metastasis is remarkably inefficient, as it requires precise coordination of each step to avoid the elimination of migrating cancer cells (Chambers et al., 2002). As an example of this principle, even though a primary tumour measuring 1 cm (approximately one billion cells) can release up to a million cells into the bloodstream daily, less than 0.1% of these cells successfully colonise distant sites due to unfavourable conditions in target tissues and failures in efficient pre-metastatic niche priming (Fares et al., 2020; Fidler, 2005; Mack and Marshall, 2010). Although inefficient, metastases may emerge long after the primary cancer has been treated. This dormant phase could result from micrometastases that remain in a non-vascularised state/state of proliferative-apoptotic balance; however, upon activation, they possess the potential to emerge into clinically detectable macrometastases when they gain the ability to develop blood vessels or recover from cell-cycle arrest (Almog, 2010; Mack and Marshall, 2010; Willis et al., 2010). Given that metastasis accounts for nearly 90% of cancer-related deaths, targeting the mechanisms of cancer cell spread and secondary tumour formation remains a critical focus of therapeutic strategies (Sporn, 1996).

Although a significant amount of progress has materialised when it comes to metastatic melanoma treatment, conventional treatment modalities such as surgical resection, chemotherapy, targeted therapies, immunotherapy and combination therapy are not without limitations. Acquired therapeutic resistance, adverse events, dose-limiting toxicity, as well as varied patient response remain integral challenges in metastatic melanoma therapy. Nevertheless, conventional therapies remain the cornerstone of melanoma management and have markedly improved survival in selected patient populations. Their application must therefore be carefully tailored on a case-by-case basis, taking into account tumour genetics, disease stage, and patient-specific factors. Examples of such therapeutic modalities are discussed further below.

### 3.4 Conventional treatments for metastatic melanoma

#### 3.4.1 Surgery

Upon observation and diagnosis of the primary tumour, surgical excision is the first immediate intervention, regardless of melanoma stage. The objective of this surgical intervention is to minimise the possibility of exaggerated localised disease as well as systemic dissemination of cancer cells to distant sites, thereby increasing the likelihood of long-term survival and minimising debilitative functional impairment (Davis et al., 2019; Eddy and Chen, 2020; Rutkowski et al., 2010; Zhang et al., 2018). Histopathological features of primary tumours are known to determine the scope of surgical removal of the primary tumour (Rutkowski et al., 2010). The rationale behind the surgical removal of metastatic melanoma is based on several factors, such as reducing the overall tumour burden and the reduction of tumour-induced immunosuppression (Hsueh et al., 2000; Tyrell et al., 2017).

#### 3.4.2 Radiation therapy

Historically, melanoma has long been considered a radiation-resistant tumour type largely due to the protective activity of intrinsic melanoma cell deoxyribonucleic acid (DNA) repair mechanisms; however, under optimal therapeutic conditions, radiation may be used as primary or adjuvant treatments (McKay and Kefford, 1995; Shi, 2017; Strojan, 2010). The universal intention of radiotherapy (RT) is to locally induce damage to cellular DNA, thereby exerting an anti-tumour effect; however, distant or systemic manifestations have been noted and reported as a consequence of the “abscopal effect”. The effect is defined as the ability of localised radiation to initiate distant and systemic anti-tumour activity through immune activation and stimulation via exposure to tumour cell antigens (D’Andrea and Reddy, 2020). Nonetheless, radiotherapy plays an important role in the management of metastatic melanoma, where it is widely employed for palliative purposes, effectively alleviating symptoms and improving patient comfort and quality of life (Mahadevan et al., 2015).

#### 3.4.3 Chemotherapy

Conventional cytotoxic chemotherapy preferentially affects rapidly dividing cells by damaging DNA or interfering with mitosis, leading to cell-cycle arrest and subsequent cell death through mechanisms such as apoptosis, necrosis, or critical impairment of the mitotic spindle (Bagnyukova et al., 2010; Morse et al., 2005; Ricci and Zong, 2006). Prior to the emergence of modern chemotherapy alternatives, there were only two United States Food and Drug Administration (FDA)-approved therapies for metastatic melanoma up until the year 2011: dacarbazine and high-dose IL-2, a cytokine serving an immunotherapeutic function (Lo and Fisher, 2014). Dacarbazine and its related compound temozolomide constitute the most commonly employed chemotherapeutic agents in melanoma disease models (Velho, 2012). Despite an efficacy level that appears relatively low, dacarbazine remains the fundamental treatment for stage IV melanoma and has profound activity in the palliative setting as well as in resistant, relapsed, or refractory metastases (Chapman et al., 1999; Eggermont and Kirkwood, 2004). When exploring additional chemotherapeutics, such as temozolomide, another alkylating agent and prodrug of dacarbazine, it is observed that temozolomide offers a viable alternative to dacarbazine for treating metastases within the central nervous system (CNS) due to its superior CNS penetration (Boogerd et al., 2007). Subsequent research using combination chemotherapy, including the Dartmouth regimen (comprising dacarbazine, cisplatin, carmustine, and tamoxifen), demonstrated an increased likelihood of toxicity whilst showing no noticeable difference in survival outcomes or treatment response rates compared with dacarbazine alone (Chapman et al., 1999).

#### 3.4.4 Targeted therapy

Advancements in targeted therapies have significantly improved the prognosis of metastatic melanoma, particularly with the introduction of BRAF and mitogen-activated extracellular signal-regulated kinase (MEK) inhibitors (Sundararajan et al., 2024). The BRAF gene encodes the B-Raf protein, a crucial member of the Raf kinase family that regulates intracellular signalling pathways involved in cell growth and survival. Specifically, B-Raf plays a key role in the MAPK/extracellular signal-regulated kinase (ERK) signalling cascade, which governs essential cellular processes such as proliferation, differentiation and secretion (Vennepureddy et al., 2016). BRAF inhibitors, including dabrafenib, vemurafenib, and encorafenib, have significantly improved tumour regression and overall survival in patients with BRAF-mutant metastatic melanoma (Chapman et al., 2011; Dummer et al., 2018; Flaherty et al., 2012). Similarly, MEK inhibitors have been shown to improve progression-free and overall survival, with the BRAF/MEK combination therapy exhibiting stronger therapeutic responses and a convenient delay in the onset of drug resistance (Flaherty et al., 2012; Robert et al., 2015a; Sundararajan et al., 2024). Another category of targeted therapy includes KIT inhibitors, such as imatinib and nilotinib, which are used in patients with specific KIT mutations and are more common in mucosal and acral subtypes (Beadling et al., 2008; Curtin et al., 2006). When activated, KIT, a tyrosine kinase, triggers the MAPK and PI3K/AKT signalling pathways, facilitating tumour progression.

#### 3.4.5 Immunotherapy

Melanoma is highly immunogenic, stemming from a substantially high mutational burden, generating tumour-associated (TAA), tumour-specific (TSA) and melanoma differentiation antigens (MDA) that are presented on major histocompatibility complex MHC class I molecules and readily recognised by CD8^+^ cytotoxic T lymphocytes (CTLs) (Alexandrov et al., 2013; Davis et al., 2019). As a result, melanoma cells downregulate TAAs and MDAs, secrete inhibitory growth factors and cytokines, and manipulate immune checkpoints. Two of the most active immune checkpoint inhibitors are antibodies against cytotoxic T-lymphocyte-associated protein 4 (CTLA-4) and programmed cell death protein 1 (PD-1) (Passarelli et al., 2017). Therapeutic blockade of CTLA-4 with monoclonal antibodies enhances T-cell activation by preventing inhibitory immune signalling and promoting anti-tumour immunity (Ribas et al., 2005). Ipilimumab, a human IgG1 anti-CTLA-4 monoclonal antibody, has demonstrated a clear clinical benefit in metastatic melanoma. A phase III trial in 502 untreated patients showed improved overall survival with ipilimumab plus dacarbazine versus dacarbazine alone (11.2 vs. 9.1 months) and higher 12-month survival rates (47.3% vs. 36.3%) (Robert et al., 2011). Similarly, PD-1 inhibitors such as pembrolizumab and nivolumab restore T-cell function and enable broader anti-tumour activity by disrupting PD-1/PD-L1-mediated immune suppression (Maverakis et al., 2015). In 2014, the FDA approved a PD-1 targeting monoclonal antibody named MK-3475, otherwise known as pembrolizumab (Hamid et al., 2013). Shortly thereafter, nivolumab was FDA-approved for metastatic melanoma. In a phase III study, 1-year overall survival was substantially higher with nivolumab (72.9%) than with dacarbazine (42.1%), while median progression-free survival more than doubled (5.1 vs. 2.2 months) (Robert et al., 2015b).

#### 3.4.6 Limitations of conventional therapy

Conventional treatment modalities have transformed the therapeutic landscape for metastatic melanoma, significantly improving patient outcomes and quality of life. Nevertheless, no current treatment strategy is without limitations, and their efficacy is often constrained by these limitations. The limitations of the above-mentioned conventional therapies are summarised in Table 2.

**TABLE 2:** A summary of the major limitations associated with conventional melanoma therapy.

Drug/Class	Major limitation (s)	References
Surgery	Ineffective once metastasis occurs	Davis et al. (2019)
Radiotherapy	Radioresistance	Gorodetska et al. (2025)
Chemotherapy	Low response rates, toxicity, resistance	Wilson and Schuchter (2016)
Targeted therapy	Primary/acquired resistance, mutation-specific	Manzano et al. (2016)
Immunotherapy	Immune-related adverse events	Robert et al. (2011)

In light of these limitations, research into medicinal-plant-based interventions has facilitated the recognition and appreciation of the diverse and abundant bioactive compounds present within, and their corresponding bioactivity. Plant-derived agents offer a promising, multifaceted approach to targeting metastatic cancer pathways while potentially helping reduce disease sequelae such as resistance and adverse effects. Accordingly, the following section will examine the pathophysiology of the metastatic cascade step by step and subsequently highlight the emerging evidence for plant-derived interventions, emphasising medicinal plants that demonstrate activity at specific stages of metastatic progression.

### 3.5 Pathophysiology of the metastatic cascade and the role of phytotherapeutic intervention in each stage

Plant biodiversity has provided human populations with a source of bioactive phytochemicals for centuries. Numerous conventional pharmaceuticals have been developed from natural product scaffolds, using compounds widely distributed in the plant kingdom. The multifaceted bioactive profiles and chemical diversity of secondary metabolites in plants can be attributed to millennia of evolutionary adaptations in response to selective pressures and diverse stimuli (Wink, 2003). It is not surprising that these adaptations translate into the production of thousands of chemical compounds within a single plant; when one considers the existence of an estimated 374,000 plant species on Earth, the total number of potential bioactives is immense (Christenhusz and Byng, 2016). Both primary metabolites, which are fundamental to normal cellular processes, and secondary metabolites, each playing distinct roles in environmental and physiological adaptation, contribute to the complex composition of phytochemicals. Secondary metabolites are often specific to particular taxonomic groups and exhibit pronounced geographical and seasonal variations, thereby exhibiting distinct biochemical profiles (Palazon and Alcalde, 2025). Investigations into the origins of small-molecule drugs have revealed that over 30% of these agents developed between January 1981 and September 2019 originated from natural products or were subsequently derivatised from them (Newman and Cragg, 2020). From a chemotherapeutic perspective, plant-derived compounds such as paclitaxel and docetaxel from the genus *Taxus*, and vincristine and vinblastine isolated from *Catharanthus roseus* (L.). G. Don., and teniposide and etoposide from *Podophyllum peltatum* L. contribute to over 60% of available anticancer therapies (Asma et al., 2022).

Medicinal plants have been extensively studied for their bioactive potential in combating various cancers, including melanoma, with promising results. These medicinal plants are known to exert their effects through multiple mechanisms, including induction of apoptosis, regulation of cell cycle progression, suppression of angiogenesis, inhibition of cell invasion, and modulation of critical signalling pathways associated with tumour growth and spread. The greater the scope of therapeutic activity, the greater the possibility of integration into a complementary or alternative therapy for melanoma aimed at preventing or reducing metastatic progression.

#### 3.5.1 Metastatic initiation, invasion and extracellular matrix degradation

Melanoma progression initially depends on sustained and uncontrolled proliferation at the primary tumour site, where malignant melanocytes undergo rapid expansion and local accumulation. The clinical significance of this early phase is apparent as continued proliferation not only increases tumour burden but also enhances the likelihood of acquiring additional alterations that enable invasive and metastatic behaviour. Intervening at this stage is therefore particularly important, as suppressing tumour cell proliferation may limit disease progression before the metastatic cascade is activated. Numerous medicinal plants have shown activity in this regard.

A study by AlQathama et al. (2022) investigated the cytotoxic effects of hexane, chloroform and methanol pod extracts of *Acacia nilotica* (L.) Willd. ex Delile (synonymous with *Vachellia nilotica* (L.) P. J. H. Hurter and Mabb.) with voucher specimen number: UQU-UCL-2016/1, on human malignant melanoma (A375) cell lines. It was determined that the chloroform and methanol extracts were the most active, with average 50% growth inhibition (GI_50_) values of 121 ± 4 μg/mL and 11 ± 6 μg/mL, respectively (AlQathama et al., 2022). Reports of the traditional use of this species for various malignancies, particularly skin cancers, have spread across India and Africa, thereby facilitating the validation of its ethnopharmacological relevance through the aforementioned scientific data (Kalaivani and Mathew, 2010). Focusing on a murine melanoma model, a 2022 study aimed to determine the antineoplastic activity of the butanol fraction of the fruit peel of *Caryocar brasiliense* A.St.-Hil. on B16F10 murine melanoma cells. The results demonstrated IC_50_ values of 390.9 μg/mL following a 24-h treatment and 226.4 μg/mL after 48 h (Silva et al., 2022). This species is primarily used for respiratory conditions and has rarely been traditionally linked to cancer or melanoma. However, its selection in this review is based on *in vitro* anti-melanoma activity and chemopreventive effects observed in murine models of preneoplastic lesions, supporting further investigation of its potential anti-cancer properties (Khouri et al., 2007; Traesel et al., 2016). In an investigation evaluating the role of *Aloe ferox* Mill. in melanoma, the gel and leaf extracts of *A. ferox* were tested for their effects on the proliferation of A375 melanoma cells (Laux et al., 2022). The gel and leaf extracts displayed IC_50_ values of 11.2 and 0.48 μg/mL, respectively. This highly active sample necessitates deeper investigation and mechanistic determination. Favourability is enhanced by the fact that this species has been used as an anti-tumour and chemopreventive folk medicine since ancient times (Chen et al., 2012). Approaching the challenge from a more phytoconstituent perspective, investigations into *Aronia melanocarpa* (Michx.) Elliott have placed much focus on the role of antioxidants in melanomagenesis, with anthocyanins from *A. melanocarpa* reducing melanoma cell proliferation in a B16F10 model in a dose-dependent manner. The IC_50_ value for the chokeberry anthocyanin-rich extract (C-ARE) was determined to be 352 μg/mL, whereas the red grape anthocyanin-rich extract (RG-ARE) exhibited a lower IC_50_ of 183 μg/mL (Diaconeasa et al., 2017). Despite being traditionally used mainly for the treatment of colds, this species has gained significant scientific attention owing to its high concentration of anthocyanins and other polyphenolic compounds with recognised chemopreventive properties. This awareness demonstrates that a medicinal plant’s pharmacological value cannot always be predicted from its ethnomedicinal use alone, thereby reinforcing the need to investigate its phytochemical composition and individual bioactive constituents when identifying new pharmacological applications (Kokotkiewicz et al., 2010). Another example of a species rich in multifaceted antimelanomagenic potential is *Buddleja saligna* Willd. An in-depth investigation examining the impact of the ethanolic leaf extract of *B. saligna* (voucher specimen number: PRU 122167) on melanomagenesis showed not only antiproliferative activity in a UCT-MEL-1 melanoma cell line but also antiangiogenic activity in an ex ovo chick embryo yolk sac membrane (YSM) assay, coupled with vascular endothelial growth factor (VEGF) inhibition. With an IC_50_ value of 33.80 ± 1.02 μg/mL in the UCT-MEL-1 antiproliferative assay, this species was shown to be more selective for cancerous cell inhibition when compared to the IC_50_ value of 54.38 ± 8.55 μg/mL against non-cancerous human keratinocytes (HaCat) (Twilley et al., 2022). Although limited documented information is available on traditional use of this species specifically for cancer or melanoma, it has long been employed in the treatment of conditions including coughs, colds, diabetes, tuberculosis, thrush, sores, anasarca and chest pain (Chukwujekwu et al., 2016). The term “sores” in ethnobotany is broad and, given the often-limited detail in historical records, may encompass a range of pathological conditions, including ulcerative lesions and abnormal tissue growths, such as metastasising melanoma. Nevertheless, the inclusion of this species in the present review is based primarily on its promising *in vitro* anti-melanoma activity and its high abundance of the pentacyclic triterpenes oleanolic acid and ursolic acid, both of which have demonstrated well-established anti-melanomagenic and anti-angiogenic properties. In the case of *Prosopis juliflora* (Sw.) DC. (synonymous with *Neltuma juliflora* (Sw.) Raf.) leaf methanolic extract (PJLME), researchers demonstrated cytotoxic activity on B16F10 and A375 melanoma cells, which displayed an IC_50_ of 20 μg/mL and 40 μg/mL, respectively (Choudhari et al., 2025). The combination of murine and human cell models in this study is favourable, as it permits the determination of the correlation between the two models and their extrapolative ability. Although the traditional use of this species does not primarily focus on antiproliferative or anticancer activity, its uses in wound healing and the treatment of various skin ailments in indigenous communities are relevant to melanoma, a malignant dermatological lesion (Thakur et al., 2024; William and Jafri, 2015). Coupled with promising activity in human cell models and available data on its acute and subacute toxicity, this species is an imperative component in demonstrating the potential of plant-derived products with anti-melanoma activity.

Examining the role of nanoparticles and phytochemical drug delivery agents aiming to expand on provisional anticancer results of saikosaponins, a team investigated the effect of ethanolic extracts of *Bupleurum chinense* DC. and a saikosaponin-d polyvinylpyrrolidone (PVP) nanoparticle formulation on the growth of A375.S2 melanoma cells with results indicating that the ethanolic extract of *B. chinense* was effective in antiproliferative activity at 250 μg/mL with an approximate 80% reduction in cell viability when compared to the control (Hu et al., 2016). When evaluated for the phytochemical inhibition of melanoma cell growth, saikosaponin-d nanoparticles proved their worth as drug delivery agents, showing a 70% reduction in cell viability at 5 μg/mL. *Bupleurum chinense* has traditionally been used for inflammatory and infectious conditions, and numerous herbal prescriptions containing this species are used to manage various cancers (Hu et al., 2016). In addition to its traditional use for cancer, considering that inflammation and immune dysregulation play key roles in melanoma development and progression, its additional traditional uses may still be relevant. Building on the potential of nanoparticles as drug-delivery agents, the expansion of *Swertia chirayita* (Roxb.) H. Karst. in the field of melanoma management was examined during a study which tested the efficacy of silver nanoparticles (AgNPs) encapsulating *S. chirayita* stem broth (decoction) against the human cutaneous melanoma cell line, SK-MEL-3. The results indicated a 50% reduction in melanoma cell viability at 15 μg/mL AgNPs. This level of activity at such low concentrations warrants further investigation of this species; however, it is important to pay careful attention to and invest in conservation initiatives and sustainable sourcing techniques, as this species has been categorised as critically endangered. There is thus a balance required between the need to search for alternative pharmacological interventions and the need to protect the phytochemical diversity present in nature by preventing destructive harvesting and promoting sustainable propagation, should product development and commercialisation proceed (Kumar and Van Staden, 2016). Although endangered, this species has been used in traditional medicine to manage a wide range of ailments, including diabetes, hypertension, ear and throat infections, fever, malaria, and, most importantly, skin disorders (Swati et al., 2023).

Examining experimental variation in bioactivity with respect to extraction temperature, another study investigated the cytotoxic potential of hot and cold *Clinacanthus nutans* (Burm. f.). Lindau (voucher specimen number: BORH 2093) leaf extracts against D24 human melanoma cells determined that after 24 h of treatment, the 100 and 200 μg/mL concentrations of the cold and hot water extracts significantly reduced D24 melanoma cell viability (*p* ≤ 0.05). The highest concentration (200 μg/mL) of the cold-water extract induced 45.7% cell death, while the hot-water extract induced 39.3% cell death, with representative EC_50_ values for both extracts exceeding 200 μg/mL (Fong et al., 2019). In an anti-apoptotic assay conducted in the same study, treatment of D24 melanoma cells with 100 μg/mL of the cold-water extract for 24 or 72 h showed high viability (>50%). In contrast, a concentration of 200 μg/mL significantly increased the number of apoptotic cells (p ≤ 0.05) at both time points, as indicated by Annexin V and 7-AAD staining. Rooted in centuries-old traditional use, this species has been extensively employed in the treatment of skin conditions and those of an inflammatory nature. In addition, numerous reports have revealed its traditional use in cancer. Combined with direct ethnopharmacological use for cancer, both inflammation and the generalised category of “skin conditions” are inherently relevant categories to melanoma, thereby justifying this species’ inclusion and promoting the need for mechanistic evaluation (Alam et al., 2016; Zulkipli et al., 2017). In another study, the antiproliferative activity of chloroform, methanol, and butanol leaf extracts of *Daphne gnidium* L. was evaluated in B16F10 and B16F0 murine melanoma cells (Chaabane et al., 2014). The chloroform extract exhibited the highest antiproliferative effect, achieving 95.19% and 82.42% inhibition on B16F10 and B16F0 cells after 48 h at 400 μg/mL, with IC_50_ values of 150 and 200 μg/mL, respectively. In contrast, the methanol and butanol extracts exhibited weaker inhibitory potential against B16F0 cell proliferation after 48 h of incubation, with IC_50_ values of 225 μg/mL and 332.5 μg/mL, respectively. In the same study, the chloroform extract at 50 and 100 μg/mL arrested B16F0 and B16F10 cells in the S phase, while 200 μg/mL induced G2-M phase arrest. Methanol and butanol extracts at 400 μg/mL also caused G2-M phase accumulation in B16F0 cells. Given that melanoma originates in the skin, the longstanding dermatological use of this species in traditional medicine may indicate bioactive properties relevant to skin health and disease (Chaabane et al., 2012). This provides a rationale for further investigation of its potential anti-melanoma activity. Even though the aforementioned data are not considered highly active, different plant parts and extraction methodologies may yield lower IC_50_ values. A study by Russo et al. (2019) evaluated the potential of the ethyl acetate bark extract of *Drimys winteri* J.R.Forst. and G.Forst. and its isolated sesquiterpene lactones, namely drimenol, isordrimenone and polygodial, in inhibiting the proliferation of A2058 and A375 human melanoma cells. The anti-proliferative IC_50_ values for the bark extract were determined to be 398 ± 0.14 μg/mL and 305 ± 0.10 μg/mL in A2058 and A375 melanoma cells, respectively. The study reported that the tested sesquiterpene lactones reduced Hsp70 expression and increased reactive oxygen species (ROS) production, which may contribute to the induction of apoptosis, highlighting their potential to regulate the tumour cell stress response. The inhibitory effect of the three tested compounds was most pronounced with polygodial, which exhibited an antiproliferative IC_50_ of 12.88 ± 0.02 µM (Russo et al., 2019). *Drimys winteri* has been used in folk medicine across the globe, with particular ethnopharmacological relevance for respiratory conditions, ulcers, fevers, skin conditions, and, most importantly in the context of this review, cancer (Filho et al., 1998). In addition to its traditional use, this plant has been extensively characterised from a phytochemical perspective, with numerous lead compounds, such as polygodial, displaying noteworthy growth-inhibitory effects in *in vitro* human melanoma models. Considering that data are available on both the *in vitro* activities of the extract and isolated phytochemicals, toxicity studies should be expanded to include preclinical and clinical stages using melanoma-relevant models. In addition, a focus on the utilisation of the same plant parts and extraction methodology across toxicity and bioactivity studies is imperative for correlating activity and characterising differences in extracted phytochemicals.

Results from a study by Peng et al. (2023) aimed at investigating the antiproliferative activity of *Graptopetalum paraguayense* (N.E.Br.) E. Walther (voucher specimen number: K000486290) against A375 melanoma cells displayed an IC_50_ value of 250 μg/mL at the 24-h time point (Peng et al., 2023). In addition, increased apoptotic cell development was observed with concurrent increases in extract concentration, with the percentage of apoptotic cells increasing significantly (*p* < 0.05) from 250 μg/mL (4% apoptotic cells) to greater than 15% at 500 μg/mL. Moreover, caspase-3 activity and chromatin condensation in A375 cells were induced by *G. paraguayense* extract at a concentration of 250 μg/mL when compared with the reference controls between the 6- and 24-h time points. Rather than exhibiting direct antimetastatic effects, this species suppressed melanoma cell proliferation, induced apoptosis and induced DNA damage *in vitro* (Peng et al., 2023). Although the concentrations required to elicit a substantial effect were relatively high in this study, available toxicity data indicate no adverse effects at doses up to 1,000 mg/kg after 28 days, with an LD_50_ greater than 1,000 mg/kg in the acute toxicity study. This promising safety profile, along with traditional uses in hepatoprotection and cardiovascular health, suggests an organ-protective effect which may be most beneficial as an adjuvant in supporting a biological system in distress from a metastatic malignant process (Chung et al., 2012; Huang et al., 2005). Another species which required slightly higher concentrations than those reported in some other comparable studies was the flower extract of *Hibiscus* x *rosa-sinensis* L. on B16F10 cell proliferation. Results indicated a 2-fold reduction in melanoma cell growth at 1 mg/mL, followed by a correlative 4-fold reduction at 2 mg/mL (Goldberg et al., 2017). Although these results pose practical challenges that necessitate administering larger doses *in vivo*, the potential of this species should not be overlooked, especially given its extensive phytochemical heterogeneity. Extracts of this species prepared using different plant parts or extractive solvents may result in markedly altered phytochemical profiles and correspondingly diverse activity. In addition, the traditional use of this species in skin disorders promotes its relevance for inclusion in this review (Amtaghri et al., 2023; Nath and Yadav, 2015). Regarding ethnopharmacology, another species with antiproliferative potential in melanoma and documented employment in the traditional management of tumours is *Ixeris dentata* (Thunb. ex Thunb.) Nakai (synonymous with *Ixeridium dentatum* (Thunb.) Tzvelev) (Yi et al., 2002). This species has demonstrated, in one study (voucher specimen number: KRIB 0000226), that the methanolic extract affected the proliferation of both cell lines investigated (A375 P and A375SM), with greater antiproliferative efficacy towards A375 P cells. At a concentration of 200 μg/mL, a 52% reduction of cell viability was observed in A375 P cells, whereas a 51% reduction in cell viability was seen in A375SM cells at the same concentration. Although more than a 50% reduction was observed at 200 μg/mL, it is important to note that at 25 μg/mL, cell viability was reduced to 57.7%, which is a noteworthy finding at such a low concentration (Lee et al., 2016). Given the observed effect at low concentrations *in vitro*, mechanistic and *in vivo* studies are warranted.

A different species, namely *Lannea coromandelica* (Houtt.) Merr., which also displayed very low active concentrations, was investigated to further consolidate understanding of its role in inhibiting B16F10 murine melanoma cells. The anti-proliferative activity of aqueous and ethanolic extracts of *L. coromandelica* (voucher specimen number: BSI/WRC/Iden.Cer./2022/1804220006437) was examined (Nargatti and Wadkar, 2024). The ethanolic extract demonstrated greater potency than the aqueous extract, with an IC_50_ of 9.69 ± 0.68 μg/mL, compared to the significantly higher IC_50_ of 75.49 ± 5.95 μg/mL observed for the aqueous extract. This study highlights the importance of considering inherent phytochemical variation when using different organic extractive solvents and their role in bioactivity. Nonetheless, both extracts were notably active at low concentrations, and given that this species has been traditionally used worldwide for the treatment of skin ailments and numerous other conditions with an immunological component, its relevance is pronounced (Imam and Moniruzzaman, 2014). Further justifying the role of extractive solvent on bioactivity, a study investigating the antiproliferative activity of *Ocimum tenuiflorum* L. revealed that in a dose-dependent manner, hexane, dichloromethane, ethyl acetate and methanol extracts of *O. tenuiflorum* leaves (voucher specimen number: BM 002) displayed significant IC_50_ values on A375 cells when compared to the positive control etoposide. The ethyl acetate and hexane crude extracts demonstrated the highest potential, with IC_50_ values of 34.79 ± 5.07 μg/mL and 36.71 ± 3.66 μg/mL, respectively, thereby emphasising their notable anti-melanoma potential (Moyo et al., 2024). In addition, this study demonstrated that *O. tenuiflorum* acted as a pro-apoptotic agent, as evidenced by characteristic apoptotic processes, including cell blebbing, nuclear fragmentation, reduced cell size, and chromatin condensation. As a testament to this multifaceted activity, it is noteworthy that this species has been used in anti-cancer applications for centuries by traditional communities, with particular focus in the Ayurvedic sphere (Hasan et al., 2023). In much the same way that the studies above demonstrated variation in bioactivity with the extractive solvent, the stage of plant development has proven to be another noteworthy contributor to variation in secondary metabolites. Building on previous studies investigating the antineoplastic activity of *Physalis peruviana* L. in adenocarcinoma, lung and colorectal cancers, aqueous fruit extracts at different stages of ripeness (voucher specimen number: UNOP8196) were investigated for their antiproliferative activity in SK-MEL-28 melanoma cells. The fruits of this species have long been recognised for their anti-cancer properties and have been used in traditional medicines, especially in South America for this particular purpose, alongside their analgesic, antiseptic and sedative properties (Puente et al., 2011). Three extracts were prepared according to the stage of fruit maturation, with E1 representing unripe fruits, E2 representing intermediate-stage fruits and E3 representing ripe fruits. It was shown that at 50 μg/mL, the E1 extract (unripe fruits) significantly decreased cell viability by approximately 20%. In the E2 fruit extract (intermediate-stage fruits) at a concentration of 50 μg/mL, a 25% reduction in cell viability was observed, which was significant compared to the control. The E3 fruit extract (ripe fruits) required higher concentrations of 1,000 μg/mL to achieve significance and a 10% reduction in cell viability (da Silva et al., 2024). Moreover, the extracts were found to enhance and upregulate the expression of caspase-9 and caspase-3. Apoptosis is often triggered by the activation of specific caspases, proteolytic enzymes that play a critical role in initiating and executing programmed cell death. Another example of the diversity of plant part selection by researchers was observed in a 2019 study, which proved valuable in ascertaining the role of *Zanthoxylum bungeanum* Maxim. seed oil extracts in the metastatic melanoma cascade. Of the five cell lines tested, the A375 melanoma cell line was the most sensitive to *Z. bungeanum* treatment, with an IC_50_ of approximately 0.37% at 24 h. Cell cycle arrest assays were performed in the same study and demonstrated that, at increasing concentrations of the extract (0.35%, 0.4%, and 0.45%), A375 cells exhibited proliferative inhibition via G1-phase cell cycle arrest (Pang et al., 2019). As a result of the increase in pro-apoptotic activity (59.75% apoptotic cells at 0.45% *Z. bungeanum* extract) and transwell migratory ability (51.16% invasion at 0.45% *Z. bungeanum* extract), it has appeared to provide an alternative which addresses multiple components of the metastatic cascade, highlighting the multifunctional potential of medicinal plants (Pang et al., 2019). Research indicates that PUFAs in seed oils play a crucial role in inducing apoptosis and inhibiting tissue invasion and subsequent tumour cell migration. It has been postulated that the high concentration of these beneficial fatty acids in *Z. bungeanum* seed oil is likely a key factor contributing to its potential anticancer activity. Given that this species has documented traditional use for cancer, further investigation into its therapeutic potential in clinical models is justified (Pang et al., 2019; Yan et al., 2023).

As shown above, extensive investigation into the antiproliferative potential of medicinal plants has yielded numerous positive outcomes. The challenge, however, is the *in vitro* nature of these assays and the difficulty of extrapolating to integrated living biological systems. Nonetheless, some *in vivo* models have demonstrated antimelanomagenic potential relevant to the proliferative stage of melanoma. An example of such a study investigated the oral administration of an ethanolic *A. melanocarpa* extract in an *in vivo* murine model (C57BL/6 mice injected with B16 melanoma cells) and discovered that chokeberry displayed significant anti-tumour effects by upregulating the development of cells which exert their antineoplastic effects via interferon-gamma (IFN-γ) production and the inhibition of macrophage and/or dendritic infiltration (Gajić et al., 2022). In another murine melanoma model, intraperitoneal injection of the ethyl acetate and n-butyl alcohol fractions from an ethanolic extract of *Euphorbia helioscopia* L. significantly reduced tumour cell proliferation (65% and 55% tumour volume reduction after 13 days at 2 mg/kg for the ethyl acetate and butanol fractions, respectively) with an effective dose of 2 mg/kg (Dou et al., 2024). In China, this species has been widely consumed orally to manage oedema, coughs and flu, tuberculosis, and cancer (Yang et al., 2021). The efficacy demonstrated above, with greater than 50% tumour reduction in both fractions tested, confirms and validates its traditional use for cancer; however, caution should be exercised when extrapolating these results to human models due to inherent differences in murine and human pathophysiology.

Not only is proliferation at the primary site an imperative contributor to metastatic initiation, but another critical early event in metastasis is also the invasion of cancer cells into surrounding tissues, which facilitates their subsequent downstream entry into the metastatic cascade (Fares et al., 2020). Framed as a hallmark of cancerous processes, the ability of a cell to alter its phenotype through a process called phenotype switching is exactly the initiator which enables them to detach from the primary tumour, degrade the extracellular matrix and migrate through adjacent tissues (Garg, 2022).

In the process of invasion, cancer cells utilise various migratory mechanisms, moving either as cohesive epithelial sheets, detached multicellular clusters, or as individual cells exhibiting mesenchymal or amoeboid morphologies (Friedl and Wolf, 2003). This degree of cellular plasticity allows tumour cells to undergo morphological and phenotypical changes throughout cancer progression, including epithelial-mesenchymal transition (EMT), collective to amoeboid transition (CAT) and mesenchymal to amoeboid transition (MAT) (Wu et al., 2021). Collective cell migration (polyclonal metastasis) is particularly prevalent in epithelial cancers such as breast, endometrial, colorectal cancers and melanoma, and is characterised by three key hallmarks namely the maintenance of intact cell-cell junctions, which ensures structural cohesion and intercellular communication; coordinated polarity and cytoskeletal dynamics across multiple cells for migratory traction force generation and ultimately, reconfiguration of the ECM together with the restructuring of the basement membrane, facilitating deeper tissue penetration and invasion (Friedl and Gilmour, 2009). In contrast, single-cell migration (monoclonal metastasis) offers a unique set of advantages, allowing cancer cells to navigate narrower spaces and potentially evade immune surveillance due to their smaller biological footprint.

**FIGURE 2:**
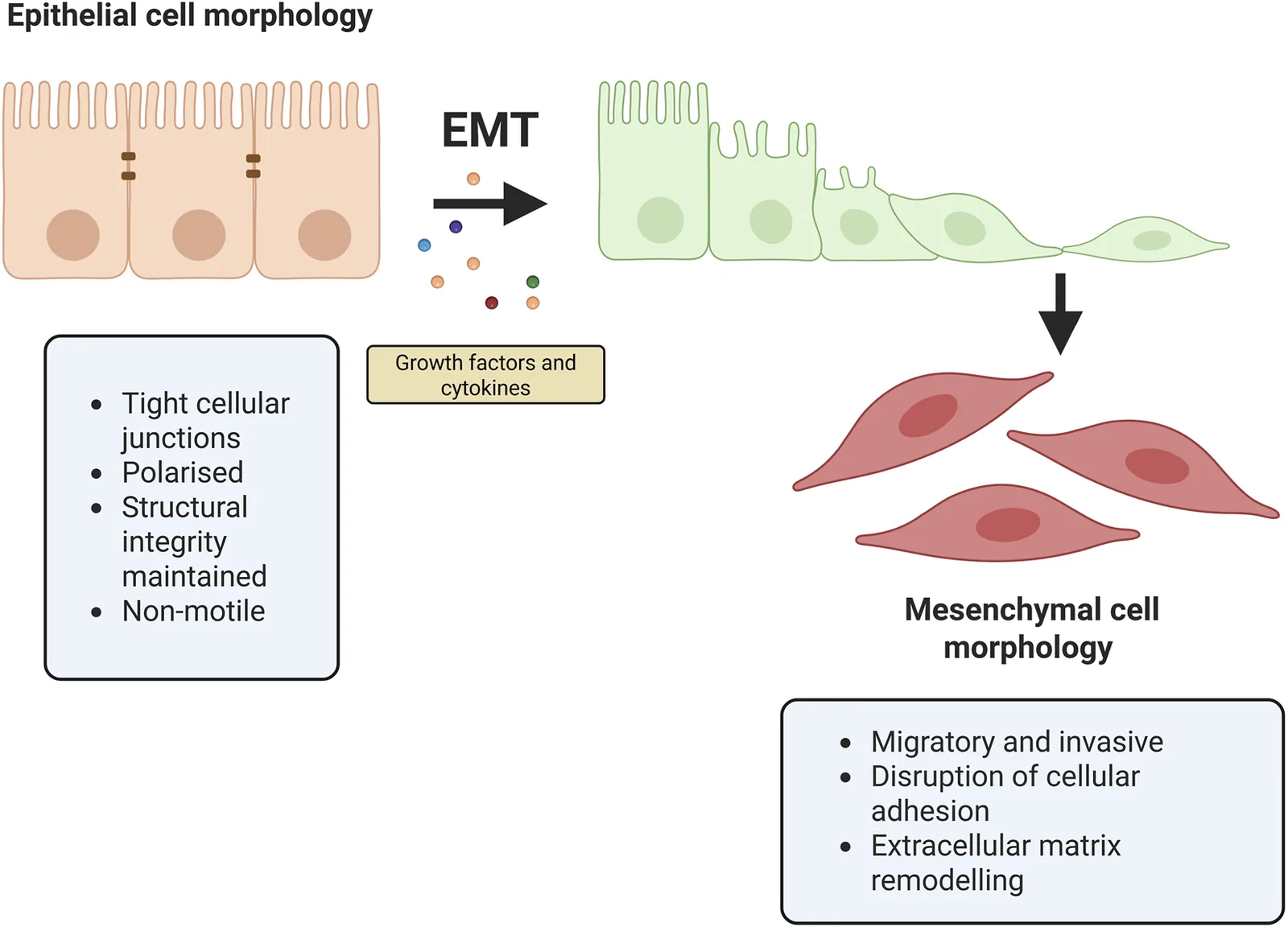
A schematic illustration of the transition from epithelial-like melanoma cells, characterised by strong cell-cell adhesion and low motility, to mesenchymal-like cells exhibiting enhanced invasiveness, motility, and resistance to apoptosis (Created in BioRender. Seaman et al. (2026) https://BioRender.com/kism9qt).

In addition to single and collective migratory methodologies, a key process occurring in both that plays a significant role in the development and progression of metastasis is the switching of the cellular phenotype to an EMT-like state (Paluncic et al., 2016). Mesenchymal-epithelial transition (MET), the inverse of EMT, is a noteworthy contributor to secondary tumour formation and a hallmark of cellular plasticity and reversibility. In the initiation of EMT, there exists an EMT transcription factor-mediated downregulation of epithelial markers leading to stabilisation of apical-basal polarity, thereby enhancing directional migration, the deterioration and destruction of epithelial cell-cell junctions, cytoskeletal reorganisation and a shift from keratin- to vimentin-type filaments, often accompanied by over-expression of N-cadherin (Lamouille et al., 2014; Nakajima et al., 2004). The morphological and structural changes that occur during EMT have been depicted in Figure 2.

Upon evaluating the role of EMT specifically in melanoma, it has been established that although melanoma cannot be classified as an epithelial cell, the adoption of its invasive, mesenchymal-like phenotype closely resembles that of cells that have undergone EMT (Li et al., 2015). It is thus not entirely unexpected that phenotypic switching could exploit the molecular mechanisms governing EMT, given that melanocytes, the precursor cells of melanoma, arise from neural crest cells that undergo EMT to delaminate from the neural tube during development (Larribère and Utikal, 2019; Mort et al., 2015). Although much focus has been placed on the role of EMT in enhancing the biomechanics of cellular migratory capacity, it is worth emphasising how EMT-driven metastasis not only enhances cellular motility and invasion but also necessitates the dynamic remodelling and degradation of the extracellular matrix.

The ECM is a highly structured and coordinated assembly of macromolecular components, including collagen, proteoglycans, glycoproteins, and enzymes, all of which play crucial roles in maintaining tissue architecture and function (Karamanos et al., 2018). Multifaceted functionality is characteristic of the ECM, which has been shown not only to provide structural support but also to exert a noteworthy influence on key metastatic processes (Paolillo and Schinelli, 2019). Beyond its supportive role, the ECM actively participates in cell signalling, specifically modulating responses to developmental, physiological and pathological cues (Rozario and DeSimone, 2010). Under normal physiological conditions, ECM homeostasis is tightly maintained to support organ development and function, thereby restoring functional integrity and tissue stabilisation; however, sustained modifications or dysregulation of the ECM can lead to pathological conditions, including fibrosis and cancer (Paolillo and Schinelli, 2019). In cancer metastasis, this tightly regulated remodelling process is captured by tumour cells to facilitate invasion and dissemination (Eble and Niland, 2019).

Extracellular matrix degradation is largely driven by MMPs, a family of zinc-dependent endopeptidases (Curran and Murray, 2000). The primary role of these enzymes is to cleave targeted ECM components such as collagen, fibronectin, and elastin, thereby promoting structural deterioration of the ECM, which had previously hindered malignant cell progression and migration. Important to note in understanding MMP activity is recognising that MMPs are secreted as inactive pro-enzymes and require extracellular activation to successfully exert their proteolytic effects (Nguyen, 2004). Beyond ECM remodelling and degradation, MMPs have proven their influence in cell survival, immune evasion and angiogenesis (Curran and Murray, 2000). Elevated MMP expression is a pillar of nearly all cancer types and has been consistently linked to poor prognosis (Bonnans et al., 2014). High levels of MMP-1 and MMP-2 are particularly associated with invasive and metastatic phenotypes, and in this light, may serve as a prognostic determinant (Väisänen et al., 1998).

Given their integral role in metastatic progression, MMPs and their subsequent inhibition represent a promising therapeutic direction. Examples of therapeutic approaches employed thus far include bryostatin compounds, modified tetracyclines, and synthetic low-molecular-weight compounds such as tissue inhibitors of metalloproteinases (TIMPs). In cancer progression, the disruption of the balance between MMPs and their endogenous inhibitors (TIMPs) facilitates ECM degradation, enabling tumour cell invasion and metastasis, whilst in normal healthy tissues, it serves as a degradative safeguard, preventing excessive ECM remodelling and tissue degradation in routine physiological processes (Lu et al., 2011; Napoli et al., 2020). Despite this promising avenue, TIMPs are known for their restrictively short half-lives and prominent side effect profiles.

As an alternative, medicinal plants such as *E. helioscopia* have demonstrated downregulation of key MMPs, including MMP-14 and MMP-15, following the administration of ethyl acetate and butanol fractions of this species to Human Umbilical Vein Endothelial Cells (HUVEC) (Ghosh et al., 2005). Although the cell line is not a melanoma model *per se*, angiogenic and invasion models outside the sphere of melanoma should not be disregarded and deemed irrelevant, as they may provide essential preliminary data to guide further investigation. The mention of the antiproliferative activity of *P. peruviana* above is further expanded on in this section as upon investigating the bioactivity of aqueous fruit extracts in various stages of ripeness, the same study demonstrated the inhibition of melanoma cell migration in all extracts at all tested concentrations with the most pronounced wound closure evident at a concentration of 250 μg/mL for E1 (40%), 1,000 μg/mL for E2 (25%) and 500 μg/mL for E3 (40%) (da Silva et al., 2024). In addition to MMP inhibition, a 2018 study aimed to address the invasive migratory component of the metastatic cascade from a melanoma perspective, utilising *Hibiscus sabdariffa* L. extract (synonymous with *Sabdariffa gossypiifolia* (Mill.) M.M.Hanes and R.L.Barrett) (Su et al., 2018). The investigators found reduced migratory capacity in B16F1 melanoma cells (concentrations of 0.4, 0.6, 0.8, and 1.0 mg/mL resulted in healing percentages of 60%, 53%, 24%, and 16%, respectively). MMP inhibition and the subsequent prevention of invasive migration via ECM degradation are key findings in natural product potential for metastatic melanoma. Subsequently, examining medicinal plants with dual antimigratory and anti-invasive potential supports the aim of developing a multifaceted intervention for metastatic melanoma that addresses numerous stages in the cascade. In addition, this study demonstrated the diverse anti-metastatic bioactivity of anthocyanins present in this species, reporting decreased tumour growth and lung metastasis *in vivo*, as well as suppressed angiogenesis. Such studies validate further investigation of this species, especially considering that cancer is not a well-documented traditional use of *H. sabdariffa*. In addition to its ethnopharmacological relevance in the management of hypertension and its laxative and diuretic properties, traditional knowledge regarding the anti-cancer activity of this species, previously undocumented, may now be augmented by these noteworthy laboratory findings (Susilawati et al., 2023). In further support of this, the alkaloid fractions from the methanolic extract of *P. juliflora* displayed anti-migratory activity on B16F10 melanoma cells in a wound healing assay, with alkaloid fraction 1 (AF1) exhibiting 45% wound closure at 10 μg/mL. In the same study, anti-invasive activity against B16F10 melanoma cells in a transwell migration assay showed significant inhibition of invasion at 10 μg/mL for AF1 and AF3. *In vivo* studies further confirmed the therapeutic potential of PJLME alkaloids for melanoma, with their ability to sensitise tumours to dacarbazine suggesting promising prospects for combination therapies (Choudhari et al., 2025). Overall, the reduction in MMP expression and migratory capacity by medicinal plants characteristically reduces the ability to disseminate, and there is thus an urgent need for intervention, as effective management in early stages, such as local invasion and migration, favours long-term survival and improved prognosis.

In summary, metastatic cell invasion involves the dynamic remodelling and degradation of the ECM; however, for tumour cells to continue proliferating at invaded and downstream sites, they require ample access to nutrients and oxygen. This is primarily achieved through angiogenesis - the formation of new blood vessels providing direct access to vascular networks and nutritive content.

#### 3.5.2 Angiogenesis

Angiogenesis, the formation of new blood vessels from pre-existing vasculature such as capillaries and post-capillary venules, is a complex and tightly regulated process representing applications in both physiological and pathological conditions. While this process has been proven essential during key physiological events such as embryonic development, wound healing and tissue remodelling, angiogenesis is also a prominent contributor to inflammatory dysregulation and subsequent malignant progression (Emmett et al., 2011). In tumours, angiogenesis ensures a continuous supply of oxygen and nutrient delivery necessary for sustained growth, while also facilitating the removal of waste products (Neitzel et al., 1999). The development of new blood vessels promotes tumour invasion and metastasis by providing pathways and access for tumour cells to enter the circulation and disseminate haematologically. The modulation of angiogenesis rests on a delicate balance between molecules with stimulatory or pro-angiogenic activity and those with inhibitory or anti-angiogenic activity. A stimulatory shift in the balance within the tumour microenvironment is imperative for melanoma tumour progression, particularly invasion and metastasis (Hanahan and Folkman, 1996). The newly established tumour microenvironment, rich in pro-angiogenic factors, triggers endothelial cell growth and directs new blood vessel formation toward the tumour via the “angiogenic switch” (Baeriswyl and Christofori, 2009).

**FIGURE 3:**
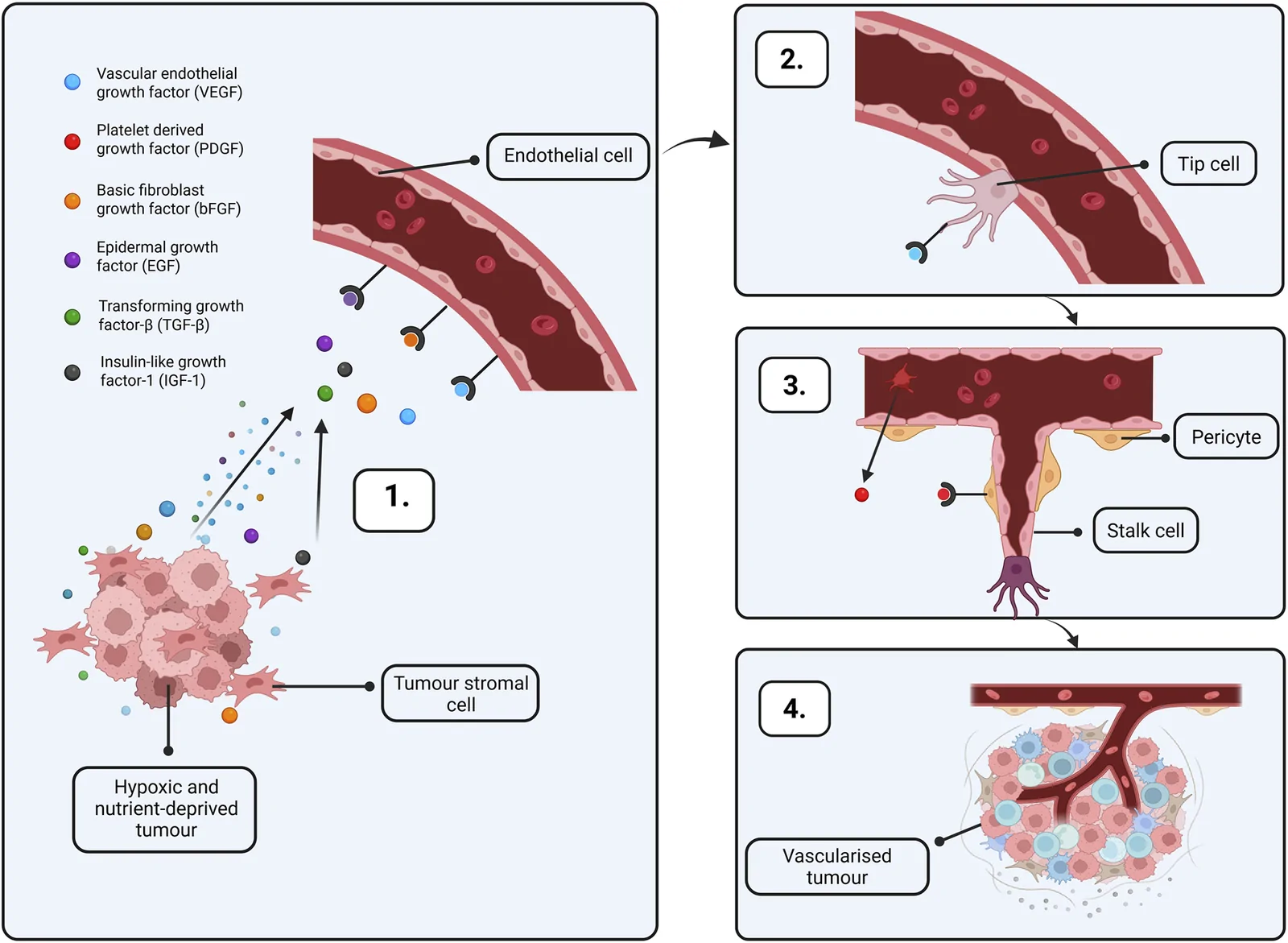
An overview of the stages of angiogenesis, from tumour hypoxia to neovascularisation, with associated growth factors and cytokines (Created in BioRender. Seaman et al. (2026) https://BioRender.com/5xqynen).

Pro-angiogenic factors exist in numerous forms, including key growth factors and cytokines. An overview of the angiogenic process, alongside a summary of the most common growth factors and cytokines involved in melanoma angiogenesis, is displayed in Figure 3.

Another growth modulator, hypoxia-inducible factor-1α (HIF-1α), is synthesised in response to local tissue hypoxia caused by nutrient and oxygen depletion induced by the proliferating melanoma. Hypoxia-inducible factor-1α induces the upregulation of VEGF-A, thereby enhancing vascular permeability and facilitating melanoma proliferation and migration (Cho et al., 2019).

Targeting the vasculature that facilitates tumour progression is a promising strategy compared with targeting cancer cells in isolation. Anti-angiogenic therapy has therefore been designated as a novel treatment approach which, if side-effect tolerability is delivered, may be universally applicable within the context of metastatic melanoma by representing a strategy to impede vascular access to secondary metastatic sites (Guo et al., 2023). Agents like bevacizumab (Avastin), ramucirumab (Cyramza), aflibercept (Eylea) and lenalidomide (Revlimid), as well as VEGF receptor inhibitors such as sunitinib (Sutent) and sorafenib (Nexavar), have shown promise in targeting VEGF activity (Malekan et al., 2024). Although early results suggested that anti-angiogenic drugs could slow or limit tumour growth in melanoma patients, these therapies have not delivered the expected long-term, effective anti-tumour outcomes. In an effort to further develop an effective and suitable therapeutic regimen, bevacizumab, the first FDA-approved anti-VEGF antibody, has been tested in combination with various chemotherapy and immunotherapy agents, including carboplatin, paclitaxel, and atezolizumab. Unfortunately, clinical trials have produced inconclusive results regarding its efficacy and safety in melanoma (Guo et al., 2023; Malekan et al., 2024).

The exploration of alternative therapies has shifted to a focus on natural approaches to angiogenic management. Angiogenesis is a multifactorial process, and areas of intervention remain extensive. As a testament to the potential of medicinal plants in anti-angiogenic strategies, both *in vitro* and *in vivo* studies confirmed the anti-angiogenic potential of *B. saligna* and DT-BS-01 (a triterpenoid mixture consisting of ursolic and oleanolic acid). *In vitro*, the inhibition of VEGF production was observed at active antiproliferative concentrations of 30 μg/mL and 5 μg/mL for *B. saligna* and DT-BS-01, respectively. *In vivo* YSM assay revealed that treatment with 15 μg/egg of *B. saligna* significantly decreased the formation of new microvessels by 53.34% ± 1.64%, compared with 57.49% ± 6.48% in the positive control group. In the case of *E. helioscopia*, in addition to tumour volume reduction, this finding was found to be in conjunction with a decrease in angiogenesis genes, which correlated well with the observation of decreased vasculature on histopathological analysis (Dou et al., 2024). Both the ethyl acetate and butanol fractions suppressed angiogenesis in Human Umbilical Vein Endothelial Cells (HUVECs), reducing tubular formation and downregulating key genes, including VEGF-B, VEGF-C, ephrin-B1 (EFNB1), and nitric oxide synthase 3 (NOS3). In addition, both compounds reduced VEGF and VEGF-B protein levels, as confirmed by immunofluorescence and Western blotting. These coordinated reductions support the anti-angiogenic activity *of E. helioscopia* even though HUVEC cells were employed as an angiogenic model and not necessarily a melanoma cell line. The value of *in vivo* models in angiogenic analysis should be considered substantial, given their ability to more accurately represent a functioning biological system. From an angiogenic perspective, a study by Su et al. (2018) demonstrated antiangiogenic activity *in vivo* using *H. sabdariffa* calyx extract at a concentration of 3 mg/mL, whereby a reduction in B16F1-induced neovascularisation was observed with a concurrent decrease in blood vessel formation (26.8%) when compared to B16 alone. This was in addition to diminished tumour volume and the prevention of pulmonary metastasis. Mechanistically, the anticancer effects appear to be mediated by suppression of MMP expression, inactivation of nuclear factor kappa B (NF-κB), and downregulation of the Rho family, Ras/MAPK, and PI3K/Akt signalling pathways, highlighting the constellation of factors influencing angiogenesis.

#### 3.5.3 Intravasation, survival in the vasculature and extravasation

Pertinent to the success of melanoma progression is the ability of cancer cells to break away from the primary tumour and invade the endothelial lining of blood vessels through a process known as intravasation. Gaining access to the vascular lumen and systemic bloodstream, dissemination is now possible. Once within the circulatory system, these detached cells are known as CTCs (Sznurkowska and Aceto, 2022). Studies using intravital microscopy have revealed that cancer cells form extensions that initially align along the cell-cell junctions of endothelial cells before penetrating between them (Roh-Johnson et al., 2014). Among the most commonly encountered variants of cellular protrusions involved in intravasation are those of invasive invadopodia, structures rich in protein components such as cytoskeletal modulators, adhesion proteins, scaffolding proteins and a host of communicative signalling molecules (Murphy and Courtneidge, 2011). Invadopodia are specialised, actin-rich protrusions formed by cancer cells that play a crucial role in the invasion of surrounding tissues. In recent years, these structures have been shown to facilitate ECM degradation and penetration, enabling tumour cells to breach the endothelial barrier and initiate intravasation into the vasculature (Paz et al., 2014). However, in order to breach the endothelial barrier, secure adhesion and attachment are required. Although results are limited to *in vitro* studies, numerous medicinal plants have demonstrated anti-adhesive activity in melanoma models. A leaf and stem extract of *Athenaea velutina* (Sendtn.) D'Arcy, with voucher specimen number: VIC40.313, extracted using a mixture of dichloromethane, chloroform, and distilled water, showed notable potential in an anti-adhesive B16F10 Matrigel assay. At concentrations of 1.56, 3.12, 6.25, and 12.5 μg/mL, the adhesive potential was reduced to 27%, 35%, 47%, and 89%, respectively. The results were deemed statistically significant when compared to the untreated control (p < 0.01) (Almeida et al., 2020). Documented traditional use of this species is extremely limited; however, it emphasises the principle that not all promising anticancer plants emerge from traditional medicine and that some are discovered through modern phytochemical and pharmacological screening programmes, such as the Brazilian forest screening investigation, which discovered the anti-adhesive activity detailed above. In a separate study, gambogic acid, isolated from *Garcinia hanburyi* Hook.f., was used to demonstrate its anti-adhesive potential in a similar B16F10 Matrigel flow adhesion assay. Gamboge resin, derived from *Garcinia hanburyi*, has traditionally been used as a haemostatic and antiseptic for wound treatment (Zhao et al., 2008). Although these uses are not directly associated with melanoma, its inclusion in this review is supported by noteworthy evidence of its *in vitro* and *in vivo* cytotoxic and antitumour activity, together with detailed phytochemical characterisation of gambogic acid, a principal bioactive constituent. Interest in this species has also grown with studies now investigating its anti-metastatic effects in melanoma models and its promising activity in combination with conventional chemotherapeutics across a range of cancers. In terms of anti-adhesive potential, when tested at concentrations of 1, 10 and 100 nM, reductions in adhesion to fibronectin of approximately 15%, 25% and 40% were observed (Zhao et al., 2008). The limitations of these assays, however, centre on the molecules they adhere to, which primarily consist of ECM components rather than the vascular endothelial wall. In an attempt to circumvent this dilemma, researchers investigated the potential of the methanol extract of *Taraxacum officinale* F.H. Wigg (synonymous with *Taraxacum* sect. *Taraxacum* F.H.Wigg.) to inhibit THP-1 monocyte adhesion to the vascular endothelium. It was found that pre-treatment with the extract at a concentration of 100 μg/mL for 1 h reduced THP-1 adhesion to the endothelium to near-baseline levels (Jeon et al., 2017). Although not conducted under melanoma-specific experimental conditions or using melanoma-specific cells, these results are a testament to the potential of medicinal plants in anti-adhesive strategies. While traditionally used for breast and uterine cancers, certain elements of malignant pathology are shared across cancer variants; therefore, this species may exhibit anti-adhesive activity in metastatic melanoma models (Singh et al., 2025). Nonetheless, should adhesion prevention fail, subsequent intravasation occurs, during which numerous complexities arise.

Intravasation, despite being classified as an inherent and pivotal contributor to haematological metastasis, is inefficient, as many tumour cells entering the bloodstream may be destroyed by the mechanical stress of laminar blood flow. Furthermore, once in circulation, cancer cells must also initiate immunological evasion and successfully implant in distant organs to form metastases (Chiang et al., 2016). Due to the intricacy of intravasation, there are few reports of medicinal plants and their ability to interfere with this process. In addition to the requirements for mechanical and immunological resilience, metastasising cells must conform to the architectural constraints imposed by tissue and tissue borders during intravasation. One such dilemma is that of nuclear squeezing, which places significant stress on the integrity of the cell’s nucleus during invasion, thereby leading to genomic instability and rearrangements, which can enhance the cell’s metastatic potential (Denais et al., 2016). Ultimately, the degree of post-intravasation survivability is determined by adaptability during circulation and extravasation.

While the majority of CTCs circulate as solitary cells, a subset forms multicellular clusters, which exhibit a significantly higher potential for metastasis formation (Aceto et al., 2014). These clusters are not solely composed of cancer cells but also incorporate stromal cells and immunological components from the original primary tumour microenvironment. The clinical relevance of this association lies in the promotion of tumour cluster heterogeneity, which favours cluster survival and has profound downstream implications for therapeutic interventions (Duda et al., 2010). Although the ability to form cellular aggregates and colonies comprised solely of melanoma cells is important, research has suggested that stromal cells in vascular transit with metastatic cell clusters provide a preliminary growth advantage to the tumour cells by creating a supportive microenvironment saturated with growth factors, cytokines and extracellular matrix components that promote cancer cell proliferation and colonisation (Duda et al., 2010).

Despite extensive morphological adaptations to dynamic environmental and structural pressures, single or aggregated CTCs reach a stage whereby the adhesive capacity of the heteroaggregate exceeds the flow velocity imposed upon them by blood flow; therefore, sectors with low hemodynamic flow, such as capillary networks and areas of slow circulation, are where CTCs most likely stabilise and extravasate (Follain et al., 2018).

Extravasation has long been viewed as a complex and restrictive step in the metastatic process; however, emerging research is challenging these established views, suggesting that stasis and adhesion can occur at multiple downstream sites (Katt et al., 2018; Nguyen, 2004). While the primary objective of metastatic extravasation is to release cancer cells into new tissues for colonisation and metastatic progression, not all CTCs follow this process. Some CTCs remain in the bloodstream, where they can continue to contribute to hematogenous dissemination, potentially reseeding either the primary tumour site or distant locations, thereby sustaining and enhancing metastatic spread. These intravascular tumour colonies have the potential to generate metastatic lesions without requiring cells to exit the vasculature (Al-Mehdi et al., 2000).

#### 3.5.4 Pre-metastatic niche priming and the formation of distant micro-metastases

Distant sites of envisioned metastasis do not merely authorise the invasion of cancer cells; rather, the process is influenced by a premetastatic niche (PMN) that is actively prepared by the primary tumour. Even before metastasis commences, the primary tumour alters the host microenvironment at distant sites, priming them to support the establishment and growth of metastatic cells. This selective modification of the microenvironment is a crucial step in enabling the secondary sites to become more conducive to cancer cell colonisation and proliferation (Peinado et al., 2017). In cases where tumour cells fail to adapt rapidly or effectively to the conditions of the local microenvironment, a subset of surviving cells may enter a dormant state. This adaptive quiescence allows cancer cells to remain viable over extended periods, during which proliferative activity is minimal and susceptibility to immune surveillance or therapeutic targeting is reduced (Giancotti, 2013). It is important to note that PMN priming differs significantly from metastatic niche establishment, as PMNs are devoid of cancer cells yet are saturated with regulatory growth factors and extracellular vesicles. Six characteristics of the PMN have been proposed and include inflammation, angiogenesis, immunosuppression, cellular reprogramming, organotropism and lymphangiogenesis, all of which enhance conduciveness to colonisation (Liu and Cao, 2016). On the other hand, metastatic niches are shaped and designed following the arrival of circulating tumour cells, in addition to the effects of surrounding regulatory determinants. Premetastatic niches are known to arise from the collective systemic impact of factors secreted by the tumour and extracellular vesicles (EVs) shed by the tumour (Peinado et al., 2017; Zomer et al., 2015).

Not only is EV transfer a prominent mediator of malignancy, but it is also essential to examine the EV content. In addition to proteinaceous and lipid EV components, which are noteworthy as communicative molecules, an important example of transferred material is messenger RNA (mRNA), which encodes genes responsible for cell migration and metastasis. Within the surrounding environment, these vesicles are taken up by nearby host cells, which, upon receiving the mRNA, begin to exhibit enhanced migration and an increased capacity to invade distant organs, even though they were not originally malignant (Zomer et al., 2015).

Extracellular vesicles are not the only means of communication used by tumour cells. In addition, exosomes, derived from diverse tumour cell populations, reshape the surrounding tissue environment to support tumour initiation and invasion. As an example, exosomes released during melanoma progression have been shown to induce vascular leakage and reprogram involved cells toward pro-vasculogenic phenotypes (Peinado et al., 2012). Concurrently, they weaken immune responses against the tumour, allowing malignant cells to spread, bypass immune detection, adhere to distant organs and foster the growth of metastases (Théry et al., 2009; Tickner et al., 2014). Due to their nanoscale size, exosomes can readily infiltrate surrounding tumour tissues and interact with cells distant from the primary tumour site, influencing both local and distant cellular behaviour. The active secretion of large quantities of exosomes delivers invasion-enhancing molecules, such as microRNAs (miRNAs), to other cancerous and non-cancerous cells, promoting their tumorigenic potential (Liu et al., 2015). Not only are the internal contents of the exosome clinically relevant, but so too are the external surface components. To achieve effective targeting, exosomes are modified through genetic manipulation of producer cells or by chemically attaching peptides to their surfaces (Bunggulawa et al., 2018; Tian et al., 2014). These techniques enable the development of biologically engineered, organ-specific exosomes that can deliver therapeutic drugs directly to metastatic sites and potentially inhibit PMN formation in pre-metastatic tissues.

Ultimately, if the PMN is successfully established, disseminated tumour cells that arrive at distant organs may survive and initiate secondary growth. At this stage, the capacity of metastasis to advance and become more aggressive depends on the ability of a single or a limited number of tumour cells to re-enter the cell cycle and undergo uncontrolled proliferation, ultimately forming a secondary tumour. In *in vitro* melanoma models investigating this particular bioactivity, colony formation or clonogenic assays are used as preliminary indicators of this potential. Using a methanolic leaf and stem extract of *Andrographis nallamalayana* J.L. Ellis (synonymous with *Andrographis beddomei* C.B. Clarke), researchers observed no colony formation in A375 melanoma cells after a 72-h treatment, but the formation of five colonies in B16F10 cells. Untreated cell controls attained 80% cloning efficacy (Purushotham et al., 2016). Although this species has limited data on its traditional use in cancer, conditions commonly treated with this plant include ulcers, snakebites and inflammation, all of which fall within the field of ailments affecting the skin/mucous membranes, with inflammation relevant to the dysregulation of immune activity observed in malignancy (Goel et al., 2021). The preliminary data above further justify its traditional use in skin conditions, in this case, malignant melanoma. In some cases, it is particularly challenging to align the traditional use of a species with the biological activity observed in laboratory tests, thereby raising the question of why it is advocated as having anticancer potential. An example of such a species with traditional uses, found in the categories of diabetes, snake bites, hepatic and renal ailments, smallpox, dyspepsia, and tuberculosis, but not cancer, is *Sarracenia purpurea* L. A study employing a B16F10 clonogenic formation model demonstrated a 99% suppression of proliferation and colony formation following pre-treatment with 100 μg/mL of the acetone stem extract of *S. purpurea* (Liu et al., 2022). Although 100 μg/mL is not considered the most highly active concentration according to established thresholds, a 99% suppression of colony formation cannot be ignored. For this reason, some previously unexplored medicinal plant candidates with poorly documented use in cancer should not be excluded from modern-day screening efforts, as identifying leads relies on a combined ethnobotanical and scientific approach. A similar trend in the reduction of clonogenicity was observed in another study, whereby even at the lowest tested concentration (20 μg/mL), the aqueous fraction of the ethanolic leaf extract of *Cotinus coggygria* Scop. significantly reduced melanoma cell clonogenicity to 20.61%. Increasing the concentration to 40 and 60 μg/mL led to near-complete suppression of colony-forming capacity (Antov et al., 2024). Ethnopharmacologically speaking, *C. coggygria* has a long history of traditional use in the treatment of skin disorders, wound healing, and cancer, making it particularly relevant to melanoma research (Marčetic̈ et al., 2013). These ethnomedicinal applications, together with emerging experimental evidence of its anticancer activity, support its inclusion as a promising candidate in evaluating plant-derived products for melanoma management. Given that *in vivo* toxicity data are available for this species, *in vivo* investigations into anti-melanoma activity should follow suit, particularly by promoting the inclusion of clinical trials and mechanistic evaluation. The demonstration of the above-mentioned *in vitro* activity at low concentrations warrants further investigation.

#### 3.5.5 Growth factors associated with metastatic melanoma

Growth factors play a critical role in the progression and metastasis of melanoma by promoting cell proliferation, evasion of exogenous regulatory mechanisms, survival, angiogenesis, and resistance to apoptosis. Dysregulation of growth factor signalling pathways, such as those involving VEGF, platelet-derived growth factor (PDGF), hepatocyte growth factor (HGF), adrenocorticotropic hormone (ACTH), insulin-like growth factor (IGF), epidermal growth factor (EGF) and fibroblast growth factors (FGFs), contributes to the aggressive behaviour of melanoma cells and their ability to invade distant tissues via metastatic progression and autonomous growth (Gibbs et al., 2021; Krasagakis et al., 1995; Metzner et al., 2011; Rodeck et al., 1991; Rodeck and Herlyn, 1991; Slominski et al., 2001; Tanaka et al., 2023).

One of the most renowned growth factors associated with melanoma and angiogenic proliferation is VEGF. Whilst expressed exclusively in melanoma cells, not in normal melanocytes, VEGF not only promotes the development of a tumour-promoting vascular network and melanoma progression but also facilitates vascular hyperpermeability. Nonetheless, the prognostic value of VEGF expression in melanoma remains largely unresolved (Gitaygoren et al., 1993; Rajabi et al., 2012). Another growth factor with angiogenic attributes similar to VEGF is PDGF (Villanueva and Herlyn, 2008). Additionally, PDGF via neuropilin-1 (NRP-1) stimulation is capable of enhancing melanoma invasiveness even in the absence of the PDGF receptor (Ruffini et al., 2017). The relationship between growth factors and their relative expression is demonstrated by EGF. Concurrent with an enhancement of melanoma proliferation, tumour-derived EGF promotes lymph node metastasis in melanoma by enhancing VEGF-C expression, whilst at the same time contributing to lymphatic endothelial activation and tumour cell motility, with elevated serum EGF levels observed in patients with sentinel lymph node micrometastases (Bracher et al., 2013).

Basic fibroblast growth factor 2 is well known as one of the most constitutively expressed growth factors in melanoma (Rodeck et al., 1991). An FGF-focused study demonstrated that supplementation with FGF-2 markedly promoted migratory behaviour in M5 human metastatic melanoma cells, whereas inhibition of cell-derived FGF-2 results in increased cellular attachment, indicating opposing roles for exogenous and endogenous FGF-2 in regulating melanoma cell motility and adhesion (Chalkiadaki et al., 2009). ACTH and its association with melanoma are supported by research indicating its involvement and expression in 70% of investigated melanomas (Kim et al., 2006). The role of this growth factor is related to the enhancement of melanocytic proliferation, melanogenesis, dendritic process formation, and immunosuppressive activity (Slominski et al., 2001). In terms of HGF, serum HGF levels have been shown to increase with melanoma progression and have demonstrated prognostic value in advanced disease, correlating with both progression-free and overall survival. Given its ability to activate the MAP kinase pathway via c-MET signalling, measuring circulating HGF may serve as a useful indicator in therapeutic assessments of melanoma (Hügel et al., 2016). A melanoma growth factor that exhibits a distinct expression profile is IGF, which, despite not being expressed by melanoma cells themselves, promotes melanoma cell survival, proliferation, and dissemination (Kanter-Lewensohn et al., 2000).

Early-stage melanomas often rely on exogenous growth signals from the tumour microenvironment, whereas advanced melanomas increasingly produce their own growth factors, enabling autocrine, endocrine and paracrine signalling that drives proliferation, angiogenesis and metastasis. Working in close association with one another, growth factors and cytokines collectively contribute an immense biochemical fingerprint to metastatic progression.

#### 3.5.6 Cytokines associated with metastatic melanoma

Critical in the progression and regulation of metastatic melanoma, cytokines modulate the tumour microenvironment, angiogenic development, melanomagenic proliferation, and significantly influence immunological responses via immunosuppression or activation. Acting via autocrine, paracrine or endocrine pathways, these small, secreted proteins play a significant role in growth regulation and cancerous progression (Lázár-Molnár et al., 2000; Richmond et al., 2009). Cytokine-based autocrine regulation involves the production of growth-stimulatory factors and the synthesis and expression of their receptors, the primary form of cytokine regulation in tumour cells. Endocrine cell regulation is characterised by haematological dissemination, bringing cytokines to distant metastatic sites. Finally, paracrine regulation is regarded as local regulation in the immediate environment surrounding the producing cell (Krasagakis et al., 1995). In the context of cancer, including melanoma, cytokines play a dual role. While certain cytokines can enhance anti-tumour immunity, others can create an immunosuppressive tumour microenvironment that facilitates tumour growth and metastasis. This variability has, in turn, made cytokines both a target and a tool in cancer immunotherapy (Dong, 2021; Kelso, 1989; Skalnikova et al., 2017). Examples of cytokines with an inhibitory nature which may prove useful in disease management strategies include IL-2, IL-12, IL-15, IL-18, IL-21, IFN- α, IFN- β, IFN-γ and granulocyte macrophage colony-stimulating factor (GM-CSF) (Bentebibel and Diab, 2021; Garbe et al., 1990; Krasagakis et al., 1995; Nicholas and Lesinski, 2011). Examples of stimulatory cytokines which contribute to advanced malignancy and disease progression include IL-1 α, IL-1 β, IL-6, IL-8 and IL-10 (Ilkovitch and Lopez, 2008).

The balance between these opposing cytokine signals significantly impacts melanoma progression and patient outcomes. Further research into the role of cytokines in metastatic melanoma has also paved the way for cytokine-based therapies that aim to harness the immune system to target and eliminate tumour cells.

In support of the potential of medicinal plants to modulate cytokines and their role in metastatic management, several studies have demonstrated promising results in inhibiting cytokines in melanoma models. In one such study, the control group in the tumour metastatic model displayed elevated levels of TNF-α, IL-6, and IL-1β (325 ± 28.2, 480 ± 40.6, and 58.2 ± 5.6 pg/mL, respectively). This was in opposition to treatment with a methanol extract of the aerial parts of *A. ferruginea* with voucher specimen number: BSI/SRC/5/23/2011-12/Tech-687, which displayed a significant reduction (p < 0.01) in the levels of the pro-inflammatory cytokines (194.6 ± 24.2, 212.8 ± 35.4, and 28.4 ± 4.8 pg/mL) in mice treated prophylactically. This species has traditionally been used for a myriad of conditions, such as leprosy and haematological disease, but in this case, its relevance lies in its sustained use for the treatment of cancer, in addition to its extensive employment in the treatment of a variety of skin conditions, justifying its inclusion in this review on the basis of ethnobotanical use. In addition to the prophylactic studies, mice with established tumours in this B16F10 model exhibited simultaneous reductions in cytokine concentrations to 269.6 ± 18.9, 342.8 ± 38.4, and 32.6 ± 4.8 pg/mL, respectively (Sakthivel and Guruvayoorappan, 2018). In a similar assay involving a Bio-Plex multiplex immunoassay system investigating IL-6, IL-8 and MCP-1 levels, administration of the ethyl acetate extract of *Moringa oleifera* Lam. resulted in significant reductions in the above-mentioned cytokine levels in tumour necrosis factor alpha (TNF-α)-stimulated cells (Luetragoon et al., 2024). The challenge, however, is that the cell line investigated was EA hy926 endothelial cells and not melanoma cells specifically. Variations in physiology among cell models pose a comparative challenge; nonetheless, this modulatory potential is imperative to address, and further investigation may yield similar activities in melanoma models. Documented traditional use of this species for cancer highlights prior awareness of its potential and encourages further exploration (Gopalakrishnan et al., 2016). Focusing specifically on melanoma, a B16F10-injected C57BL/6 mouse model revealed that administration of *Piper longum* L. extract reduced IL-1β from 30 ± 3.7 to 21.7 ± 3.8 pg/mL after 9 days of treatment. Similarly, IL-6 was reduced from 320 ± 9.5 to 69.4 ± 5.8 pg/mL, TNF-α from 630 ± 8.5 to 185.9 ± 9.2 pg/nL and VEGF from 150 ± 12 to 79 ± 12.4 pg/mL. The reduction in VEGF concentration correlated directly with reduced tumour-directed capillary formation (Sunila and Kattan, 2006). Although this species has been used traditionally for a wide range of ailments, most commonly cardiac conditions, digestive disorders, arthritis, pain, and respiratory tract infections, reports describing its use in cancer are comparatively limited (Lal et al., 2025; Sunila and Kuttan, 2006). Consequently, its inclusion in this review is supported primarily by its favourable safety profile and promising *in vivo* anti-melanoma activity rather than its traditional medicinal applications.

#### 3.5.7 The role of melanotransferrin in melanoma

Functioning as a 97-kDa key protein of the transferrin family, melanotransferrin, also known as human melanoma-associated antigen p97, shares unique characteristics with its transferrin, lactoferrin and ovotransferrin counterparts, including notable amino acid sequence homology and iron-binding abilities, despite exhibiting a questionable contribution to iron homeostasis (Bertrand et al., 2007). Melanotransferrin exists in two forms, namely the membrane-bound form and the soluble secreted form. Cell surface anchorage by a glycosylphosphatidylinositol (GPI)-anchor is characteristic of membrane-bound melanotransferrin (Alemany et al., 1993; Bertrand et al., 2007). Melanotransferrin has been shown to be significant in the epidemiology and progression of metastatic melanoma.

A 2006 murine study demonstrated how the downregulation of melanotransferrin expression resulted in the *in vitro* reduction of metastatic proliferation and migration, as well as tumorigenic inhibition *in vivo* (Dunn et al., 2006). Additionally, investigations focusing on the cessation of melanotransferrin expression and hyperexpression of melanotransferrin using five novel models in melanotransferrin knockout mice found that hyper-expression correlated directly with accelerated cellular growth and development, whereas expression reduction produced the opposite result (Suryo Rahmanto et al., 2007). Correlating with this data, a study measuring the small interfering RNA (siRNA)-induced downregulation of melanotransferrin expression in SK-MEL-28 melanoma cells found a lesser degree of metastatic cell invasion, migration and cell surface activation in melanotransferrin-silenced cells (Bertrand et al., 2007).

From an angiogenic perspective, researchers demonstrated that melanotransferrin exposure can elicit responses that mimic those to FGF-2 and VEGF-1. Introduction of recombinant serum melanotransferrin into chorioallantoic membrane (CAM) subjects elicited macroscopic and microscopic increases in vascular density, whereas in the Boyden chamber assay, recombinant melanotransferrin facilitated enhanced chemotactic migration of human microvascular endothelial cells (Sala et al., 2002). Although considerable time and resources have been devoted to unravelling the role of melanotransferrin in metastatic melanoma, discrepancies remain regarding the precise physiological and biochemical functions and the involvement of transferrin homologs. One study showed that melanotransferrin may inhibit neovascularisation in SK-MEL-28 melanoma cells; however, conflicting findings exist, as previous studies have demonstrated that serum melanotransferrin is pro-angiogenic and promotes plasmin production. It has been hypothesised that this observation may be explained by the antagonisation of pro-angiogenic effects by membrane-bound melanotransferrin. (Michaud-Levesque et al., 2007; Sala et al., 2002).

Nonetheless, the somewhat unresolved function and importance of this unique protein have prompted limited investigation into its exact mechanism of action and its role in metastatic progression. Subsequently, there are few to no reports of the role of medicinal plants in addressing melanotransferrin as a therapeutic target.

### 3.6 Phytochemicals with known anti-melanoma activity and their potential anti-metastatic function

Recently, there has been an accumulating body of evidence indicating that medicinal plants such as those described above have demonstrated inhibitory activity across key stages of the metastatic cascade, predominantly using crude or semi-purified extracts. Proven bioactivity in proliferation, migration, invasion, angiogenesis, and EMT modulation supports their potential as multi-target anti-metastatic agents. However, while whole extracts often exhibit synergistic activity due to dynamic interactions among phytochemicals, the contributions of individual phytochemicals should not be overlooked. Isolated compounds offer numerous alternatives towards the design of products with more targeted mechanistic activity, improved reproducibility, precise dose control, and greater ease of standardisation. Favourably, these factors are imperative for therapeutic development, optimisation and translational potential. Phytochemicals with proven activity in the field of melanomagenesis have been described below in Table 3.

**TABLE 3:** Bioactive phytochemicals against melanoma and the metastatic cascade.

Phytochemical name and example of plant origin	Stage(s) of metastasis targeted	Model type	Biological activity	*In vivo* toxicity data available	Clinical evidence available for melanoma	References
22*β*-hydroxytingenone (22HTG) - Quinonemethide triterpene *Salacia impressifolia* (Miers) A.C.Sm	• Proliferation (Cytotoxicity, cell cycle, apoptosis, ROS generation, gene expression) • Invasion	• Proliferation: *In vitro* cytotoxicity • Invasion: Reconstructed 3D skin model	IC_50_ of 3.2 µM against SK-MEL-28 cells after 72 h exposure. Transcript levels of BRAF, NRAS, and KRAS genes were significantly inhibited after 24 h exposure when compared with the negative control (*p* < 0.05). Treatment with 5.0 μM 22-HTG showed a reduction in invasion points in the 3D skin model	No	No	Aranha et al. (2021)
Affinisine - Indole alkaloid *Tabernaemontana catharinensis* A. DC.	• Proliferation (Cytotoxicity, cell cycle, apoptosis)	• *In vitro* cytotoxicity	Antiproliferative IC_50_ values ranged from 48.22 ± 1.74 to 75.04 ± 4.03 μg/mL at 24 h, and 32.86 ± 2.54 and 57.69 ± 3.94 μg/mL at 48 h for A375, WM1366 and SK-MEL-28 cells, respectively. Treatment of A375 cells with 57 and 65 μg/mL resulted in 58.16% and 87.16% total apoptotic cells, respectively. In WM1366 cells, treatment at 32 and 45 μg/mL significantly increased apoptosis, with 11.37% and 20.06% apoptotic cells, respectively. SK-MEL-28 cells treated with 46 and 55 μg/mL showed 38.83% and 60.69% apoptosis, respectively	No	No	Rosales et al. (2019)
Apigenin - Flavone *Prosopis juliflora* (Sw.) DC.	• Proliferation (Cytotoxicity, cell cycle, apoptosis) • Migration • Invasion	• Proliferation: *In vitro* cytotoxicity • Migration: *In vitro* wound healing assay • Invasion: *In vitro* transwell assay	Inhibited the proliferation of A375 and C8161 cells with IC_50_ values of approximately 100 µM and 120 μ M, respectively. It completely blocked cell migration at 100 µM (24 h) and invasion at 80 µM	No	No	Zhao et al. (2017)
Arnicolide D - Sesquiterpene lactone *Centipeda minima* (L.) A.Braun and Asch	• Proliferation (Cytotoxicity, cell cycle, apoptosis)	• Proliferation: *In vitro* cytotoxicity	Treatment with 4 µM Ar-D increased the G2/M phase cell population by 129% compared to controls, displayed a reduction in A375 cell viability to 25% and induced apoptosis in A375 cells, raising the apoptotic rate from 5.2% to 22.5%, with inhibition of NF-κB signalling	No	No	Zhu et al. (2019)
Cannabidiol - Cannabinoid *Cannabis sativa* L	• Proliferation (Tumour size and survival rate)	• Syngeneic mouse model	After 14 days of treatment, the tumour size in the control group was 1,236 ± 295 mm^3^, while the group treated with cannabidiol had a tumour size of 823 ± 200 mm^3^. *In vivo,* the control group demonstrated a survival time of 14.8 ± 0.84 days, whereas the group treated with CBD demonstrated a survival time of 19.0 ± 2.0 days. C57BL/6 mice injected with B16F10 murine melanoma cells were used	Yes	No	Simmerman et al. (2019)
Carvone – Terpenoid *Daphne gnidium* L	• Proliferation (Cytotoxicity, cAMP signalling)	• *In vitro* cytotoxicity and cAMP ELISA	At 200 µM, approximately 30% reduction in cell viability occurred in B16F10 melanoma cells and was statistically significant when compared to the DMSO vehicle control. At the same concentration, cAMP signalling was activated	Yes	No	Kang et al. (2020)
Catechin – Polyphenolic *Acacia nilotica* (L.) Willd. Ex Delile	• Invasion	• Syngeneic mouse model	An 82.2 % reduction in metastatic lung nodules was observed when compared to the control and was determined statistically significant (p < 0.001) in B16F10 injected C57BL/6 mice	Yes	No	Menon et al. (1995)
Chlorogenic acid - Phenolic *Aronia melanocarpa* (Michx.) Elliott	• Proliferation (Cytotoxicity)	• *In vitro* cytotoxicity	At 30 μ M, B16 cellular proliferation was reduced by approximately 7% when compared to the 100% growth control	Yes	No	Li et al. (2014)
Chrysoeriol - Flavonoid *Echinacea purpurea* (L.) Moench	• Proliferation (Cytotoxicity, apoptosis and protein expression)	• *In vitro* cytotoxicity and Western blot	Chrysoeriol induced apoptosis in A375 cells at 30 μM (5.5%) and 40 μM (10.6%), and in B16F10 cells at 30 μM (6.2%) and 40 μM (11.2%). Additionally, chrysoeriol (10, 20 and 30 μM) dose-dependently reduced the protein levels of phospho-STAT3 (Tyr705) and phospho-STAT3 (Ser727) in both A375 and B16F10 cells	No	No	Liu et al. (2023)
*Codonopsis lanceolata* polysaccharide (CLPS) - Polysaccharide *Codonopsis lanceolata* (Siebold and Zucc.) Benth. and Hook.f. ex Trautv	• Proliferation (Metastatic nodule formation) • Migration	• Proliferation: Syngeneic mouse model • Migration: *In vitro* transwell assay	In a B16F10 pulmonary metastasis model, CLPS reduced pulmonary metastasis by 21.8% at 200 mg/kg and by 37.7% at 400 mg/kg. CLPS inhibited migration of B16F10 cells, as evidenced by reduced migratory distances of 112.5 μm at 200 μg/mL and 76.2 μm at 400 μg/mL, compared to control cell migration of 135.5 μm	No	No	Liu et al. (2017)
Daphnetin - Coumarin *Daphne gnidium* L	• Proliferation (Cytotoxicity and synergy)	• *In vitro* cytotoxicity	At 200 µM, reductions in cell viability of approximately 80%, 55%, 90% and 60% in FM55 P, A375, FM55M2 and SK-MEL-28 cell lines, respectively. Synergistic interactions noted with conventional cytostatic drugs	Yes	No	Wróblewska-Łuczka et al. (2023)
Daphnoretin – Coumarin *Daphne gnidium* L	• Proliferation (Cytotoxicity, apoptosis)	• *In vitro* cytotoxicity	Displayed antiproliferative IC_50_ values of 37.81 and 53.46 μg/mL against A375 and B16 melanoma cells. At 60 μg/mL, daphnoretin increased the apoptotic rate in A375 cells to 20% when compared to the control at 5%. Dose-dependent increase of ROS in A375 cells after 24 h of treatment	Yes	No	Wang et al. (2021)
Ellagic acid - Polyphenol *Hibiscus* x *rosa-sinensis* L	• Proliferation (Cytotoxicity)	• *In vitro* cytotoxicity	Ellagic acid inhibited the growth of 1205Lu cells at 50 and 100 μM at 24 h, and WM852c cells at 25, 50, and 100 µM. This growth inhibition persisted over 72 h across all tested concentrations (25–100 µM)	Yes	No	Jensen et al. (2011)
Eugenol - Phenolic *Ocimum tenuiflorum* L	• Proliferation (Tumour volume and metastatic nodules)	• B16 xenograft study	A 19% tumour growth delay was observed in the test group treated with eugenol when compared to the control. Reduction of 40% in tumour size was demonstrated, in addition to no observed metastatic nodules/signs of invasion, compared to the control, of whom 50% of subjects developed metastasis	Yes	No	Ghosh et al. (2005)
Gallic acid – Phenolic acid *Kalanchoe fedtschenkoi* Raym.-Hamet and H.Perrier	• Proliferation (Cytotoxicity)	• *In vitro* cytotoxicity	Gallic acid-enriched fractions displayed cytotoxicity against B16F10 melanoma cells with an IC_50_ of 43.0 μg/mL and an IC_50_ of 93.6 μg/mL against MV3 melanoma cells	Yes	No	Casanova et al. (2024)
Ginsenoside Re – Triterpenoid saponin *Panax ginseng* C.A.Mey	• Proliferation (Cytotoxicity, tumour volume)	• *In vitro* cytotoxicity and B16F10 xenograft model	At a concentration of 12.5 μM, cell viability in B16F10 cells was reduced to approximately 90%, and at 200 μM, cell viability was approximately 75%. B16F10 xenograft model revealed a dose-dependent reduction in tumour volume	Yes	No	Hwang et al. (2023)
Monogalactosyl diacylglycerol 1 (MGDG-1) - Galactolipid *Impatiens parviflora* DC.	• Proliferation (Cytotoxicity, synergistic activity)	• *In vitro* cytotoxicity	MGDG-1 exhibited strong cytotoxicity against A375 melanoma cells, with IC_50_ values of 31.64 μg/mL at 24 h and 15.14 μg/mL at 48 h. Mix3 (MGDG-1 at 44.5 μg/mL and doxorubicin at 0.6 μg/mL) reduced A375 melanoma cell viability to 97%, demonstrating twice the potency of doxorubicin when used alone	Yes	No	Grabowska et al. (2021)
Methyl *p*-coumarate – Phenylpropanoid ester *Trixis michuacana* Lex	• Proliferation (Cytotoxicity)	• *In vitro* cytotoxicity	Methyl p-coumarate reduced B16F10 cell viability in a dose-dependent manner, with an IC_50_ of 130 μM (23.2 μg/mL)	Yes	No	Satooka et al. (2022)
Moscatilin – Bibenzyl derivative *Dendrobium loddigesii* Rolfe	• Proliferation (ROS generation, protein expression)	• *In vitro* ROS assay and Western blot	At 12.5 µM, Hsp70 expression was reduced to between 0.4 and 0.6 arbitrary densitometric units with apoptotic inhibition and ROS generation activity	No	No	Cardile et al. (2020)
Oleandrin – Cardiac glycoside *Nerium oleander* L	• Proliferation (Gene expression, apoptosis, cytokine expression)	• qRT-PCR, TUNEL apoptosis assay, IFN-γ ELISA	Oleandrin (47 nM) reduced hsa-miR-146a-5p and hsa-miR-21-5p expression in A375 cells, induced significant apoptosis (43.64% ± 10.1%) and decreased IFN-γ levels by approximately 2-fold– 33.39 ± 3.5 ng/mL	Yes	No	Eroğlu Güneş et al. (2022)
Oleanolic acid - Triterpenoid *Buddleja saligna* Willd	• Proliferation (Cytotoxicity) • Angiogenesis	• Proliferation: *In vitro* cytotoxicity • Angiogenesis: In ovo chick chorioallantoic membrane (CAM) assay	Demonstrated an IC_50_ value of 13.08 ± 3.03 μg/mL against UCT-MEL-1 cells. At 30 µM, vascular density was substantially reduced in the CAM assay	Yes	No	Caunii et al., (2017), Twilley et al. (2022)
Palmitic acid – Fatty acid *Swertia chirayita* (Roxb.) H.Karst	• Proliferation (Cytotoxicity)	• *In vitro* cytotoxicity	25% reduction in B16F10 murine melanoma cell viability at 200 μ M, with significant DNA fragmentation in SK-MEL-28 cells at the same concentration	Yes	No	de Sousa Andrade et al. (2005)
Phyllacanthone - Bis-*nor*-diterpene *Cnidoscolus quercifolius* Pohl	• Proliferation (Cytotoxicity) • Migration	• Proliferation: *In vitro* cytotoxicity • Migration: *In vitro* wound healing assay	Exhibited moderate cytotoxicity in chemoresistant A2058 melanoma cells, with an IC_50_ value of 40.9 μM. A2058 cells treated with 50 μM phyllacanthone exhibited reduced cell migration into the wound area over 3–24 h compared with untreated cells (*p* < 0.05)	No	No	de Oliveira-Júnior et al. (2022)
Polygalatenoside A - Saponin *Dudleya brittonii* Johanss	• Proliferation (Cytotoxicity)	• *In vitro* cytotoxicity	Treatment with 0.3–3 μM polygalatenoside-A inhibited B16F10 cell proliferation by 20%–50%	No	No	Kim et al. (2022)
Polygodial - Sesquiterpene lactone *Drimys winteri* J.R.Forst. and G.Forst	• Proliferation (Cytotoxicity)	• *In vitro* cytotoxicity	Cell viability inhibition with IC_50_ values of 12.88 ± 0.023 and 18 ± 0.02 μg/mL in A375 and A2058 melanoma cells, respectively	No	No	Russo et al. (2019)
Quercetin - Flavonoid *Lannea coromandelica* (Houtt.) Merr	• Proliferation (Cytotoxicity) • Migration • Invasion	• Proliferation: *In vitro* cytotoxicity • Migration: *In vitro* wound healing assay • Invasion: *In vitro* transwell assay	Antiproliferative activity, as indicated by IC_50_ values of 99.59 ± 10 and 118.1 ± 14.2 µM against A375 and A2058 melanoma cell lines, respectively. At 40 µM, A375 and A2058 cell migration and invasion were strongly inhibited	Yes	No	Cao et al. (2014)
Rosmarinic acid – Caffeic acid ester *Ocimum tenuiflorum* L	• Proliferation (Cytotoxicity) • Migration • Invasion	• Proliferation: *In vitro* cytotoxicity • Migration: *In vitro* wound healing assay • Invasion: *In vitro* transwell assay and Western blot	Reduction in A375 cell proliferation by approximately 50% at 200 μg/mL after 48 h, with a 75% reduction in migration (relative migration distance = 0.25) and a 70% decrease in relative invasive cell rate (0.3) observed at the same concentration. Relative MMP-9 expression value of 0.4 compared to control of 1.0 at 200 μg/mL	Yes	No	Huang et al. (2021)
Saikosaponin-d - Saponin *Bupleurum chinense* DC.	• Proliferation (Cytotoxicity)	• *In vitro* cytotoxicity	At a concentration of 10 µM, cell viability in A375.S2 melanoma cells was reduced by 90% using a nanoparticle formulation of saikosaponin-d	Yes	No	Hu et al. (2016)
Tetrandrine – Bisbenzyl isoquinoline alkaloid *Stephania tetrandra* S. Moore (synonymous with *Botryodiscia tetrandra* (S.Moore) L.Lian and Wei Wang)	• Proliferation (Cytotoxicity, tumour volume)	• *In vitro* cytotoxicity and *in vivo* A375 xenograft model	Antiproliferative IC_50_ values of 10.82 ± 0.67 μM, 11.27 ± 0.42 μM, 9.07 ± 0.004 μM against A375, MV3 and SK-MEL-28 melanoma cells, respectively. Reduced tumour volume was observed in the *in vivo* subcutaneous transplantation mouse xenograft model	Yes	No	Ji et al. (2024)
Ursolic acid – Triterpenoid *Buddleja saligna* Willd	• Proliferation (Cytotoxicity)	• *In vitro* cytotoxicity	At 100 µM, an 87.34 ± 5.50% reduction in SK-MEL-2 cellular proliferation was observed with a subsequent IC_50_ value of 58.43 µM. In a separate study, ursolic acid exhibited an IC_50_ of 2.31 ± 0.54 μg/mL in UCT-MEL-1 cells, with a selectivity index greater than 1 compared with noncancerous HaCat human keratinocyte cells	Yes	No	Caunii et al. (2017), Twilley et al. (2022)
Withaferin-A – Steroidal alkaloid *Withania somnifera* (L.) Dunal	• Proliferation (Tumour volume and metastatic nodules)	• Syngeneic mouse model	At 8 mg/kg, withaferin-A reduced melanoma tumour volume to approximately 18 mm, with a concurrent decrease in metastasised lung tumour nodules to 20, both of which were statistically significant compared to the control in a B16F10-induced lung metastatic model	Yes	No	Sherapura et al. (2025)

## 4 Established models of evaluation in melanoma studies and practical considerations of phytotherapy

### 4.1 Evaluation models in melanoma

#### 4.1.1 *In vitro* 2D cell culture models

2D monolayer cultures remain among the most widely used experimental platforms for preliminary screening and initial evaluation of anti-melanoma activity. Such models commonly employ cell lines, such as human melanoma cell lines A375, SK-MEL-28, and WM115. In terms of the experimental process, these models involve the culture of melanoma cells as adherent monolayers on plastic substrates, allowing controlled investigation of cellular responses to plant extracts or fractions (Beaumont et al., 2014). The murine B16 series, particularly B16F10, is frequently used in mechanistic and metastatic studies due to its high invasive and metastatic potential. Considering the fact that it is often demonstrated how little activity *in vitro* translates to limited potential in clinical trial evaluations, 2D models are therefore immensely beneficial for high-throughput preliminary screening prior to the involvement of more advanced and comprehensive preclinical evaluations (Rebecca et al., 2020).

Commonly employed techniques in the field of 2D melanoma cell culture models include cell viability assays, apoptosis analyses, cell cycle profiling, reactive oxygen species detection, and molecular assessments via Western blotting or quantitative polymerase chain reaction (qPCR) to evaluate modulation of signalling pathways. In addition, functional assays such as wound healing via scratch assay analysis, transwell migration representative of ECM degradation and invasion assay and clonogenic assays are routinely utilised to examine the antimelanomagenic potential of medical plant extracts (Eves et al., 2000; Konieczkowski et al., 2014; Rebecca et al., 2020; Satyamoorthy et al., 2002). Collectively, these models serve as essential early-stage tools for identifying promising bioactive extracts, establishing IC_50_ values, and elucidating underlying molecular mechanisms before progressing to more complex experimental systems.

The principal advantages of 2D models lie in their affordability, reproducibility, ease of standardisation, and suitability for determining preliminary dose-responses. The fact that 2D melanoma cell culture models are investigated under highly controlled conditions that can be easily manipulated enables rapid comparison among multiple plant extracts, fractions, or pure compounds. However, important limitations must be acknowledged. Although 2D culture systems are technically straightforward and have made significant strides in our understanding of melanoma biology, they unfortunately do not effectively reflect the structural and functional complexity of tumour biology and its integration into complex biological systems. Growing as 2D monolayers rather than in matrices representative of 3D architecture limits the representation of tumour-associated changes in the physiological and structural properties of melanoma cells. In addition, rapid proliferation rates *in vitro*, as well as genetic drift observed in long-term passaged cells, pose interpretational complexities and translatability to *in vivo* models (Rebecca et al., 2020). Furthermore, numerous features of solid tumours and systems biology, such as cumulative immune involvement, hypoxic gradients, ECM interactions, signalling and cell-cell communication, and stromal support processes, are absent in 2D models (Marconi et al., 2018). Furthermore, there is an inherent failure to reflect realistic drug penetration under simplified monolayer conditions, as well as to capture tumour heterogeneity and pharmacokinetic influences according to the principles of ADME, which have limited applicability in 2D models. This may subsequently result in an overestimation of their therapeutic efficacy compared with more physiologically relevant models (Das et al., 2015).

#### 4.1.2 *In vitro* 3D cell culture models

Three-dimensional (3D) culture systems have emerged as more physiologically relevant *in vitro* platforms for studying melanoma biology and evaluating therapeutic agents, including phytotherapeutic candidates. Unlike conventional 2D monolayers, 3D models permit the growth of melanoma cells in multicellular aggregates or within scaffold-based matrices, thereby mimicking tumour architecture to a far greater extent. It is important to note that key features of complex cellular aggregates and solid tumours, such as cell-cell adhesion, spatial organisation, ECM interactions and oxygen and nutrient gradients, may be more effectively replicated in such models (Lin et al., 2022; Smalley et al., 2008).

One primary example of a 3D *in vitro* approach is the employment of melanoma spheroids, which are defined as 3D aggregates of tumour cells that exhibit greater potential in replicating the structural and functional complexity of solid tumours (Lin and Chang, 2008). Spheroids are known to be more representative of nutrient and oxygen gradients, and as such, often develop a hypoxic core and display heterogeneous growth patterns (Raza et al., 2017). Cells located at the periphery of the aggregate are highly proliferative, whilst those centrally localised exhibit a less proliferative phenotype. Spheroids and their development over time represent different growth phases in melanoma. Early spheroids are representative of the radial growth phase, whereas metastatic-derived spheroids mimic the vertical growth phase and can be used to assess therapy responses (Rebecca et al., 2020).

A particularly unique feature of spheroid utilisation is the potential for co-cultivation with other cell types to enhance physiological relevance and the importance of tumour-stroma interactions. Incorporating additional specific cell populations allows researchers to model distinct features of the tumour microenvironment and better understand their influence on tumour behaviour (Nomdedeu-Sancho et al., 2023). One such study demonstrated how, for instance, when melanoma spheroids embedded in a collagen matrix were co-cultured with fibroblasts, the fibroblasts displayed enhanced infiltration of the tumour mass, ultimately converting into cancer-associated fibroblasts (CAFs). The compounding dilemma then originates when these CAFs secrete ECM proteins and growth factors, which in turn provide both chemical and architectural support that enhances tumour survival and can promote resistance to chemotherapeutic agents (Flach et al., 2011). Despite their advantages in mimicking tumour architecture and microenvironmental gradients, melanoma spheroid models are primarily limited by their initial derivation from 2D cultures, which may not fully recapitulate *in vivo* phenotypes. Additionally, it has been demonstrated that spheroid aggregates typically allow only a limited number of predetermined cell types to be co-cultured and are not easily maintained over long periods for longitudinal analyses. This constraint hinders their utility for evaluating chronic exposure to phytochemicals (Rebecca et al., 2020).

An alternative to melanoma spheroids is the utilisation of 3D skin reconstruct models, which provide a contextually relevant platform that mirrors the architecture and cellular interactions of human skin. Comprised of a stratified epidermis containing keratinocytes and melanocytes, these dynamic skin models also possess a functional basement membrane and a dermis with fibroblasts embedded in collagen type I (Li et al., 2011). Due to their morphological and functional organisation, 3D human skin reconstructs allow investigation of physiologically relevant cell-cell and cell-matrix interactions, including autocrine and paracrine signalling between melanoma cells, keratinocytes, and fibroblasts (Li et al., 2011). When incorporated into these skin constructs, melanoma cells exhibit behaviours that closely reflect tumour progression and aggressiveness seen in patients, making the model highly relevant for studying early-stage tumour biology.

Despite the complexity and advantages of skin reconstruct models in melanoma, it has been emphasised how these models are hindered by organisational and technical challenges. These include the requirement for continuous monitoring over prolonged culture periods, which makes it a real challenge to ensure consistency and validity. In addition, technically competent staff are required due to the advanced nature of the model and the requirement for consistent maintenance and evaluation (Beaumont et al., 2014). Therefore, a combination of spheroid modalities with skin reconstruct models in melanoma is essential in highlighting how the two model systems serve complementary roles in preclinical phytotherapy research. Spheroids facilitate high-throughput screening; however, the contextual link to the skin is largely lost, and this link can be better reproduced in skin reconstruct models.

#### 4.1.3 Xenograft models

Exploring more in-depth preclinical evaluations, xenograft models represent an important pillar of melanoma research and provide an opportunity to link *in vitro* findings to clinical translatability. In these models, human melanoma cells, which have either been acquired through long-established cell lines or through primary tumour samples, are subsequently implanted into immunocompromised mice (commonly nude, NOD/SCID, or NSG strains) (Ny et al., 2020). The benefit of a physiologically integrated setting is evident following successful grafting, which permits the establishment of dynamic interactions between melanoma cells and stromal components, lymphatic networks and vasculature, as well as enabling the determination of invasion, angiogenesis and tumour growth kinetics (Beaumont et al., 2014). As mentioned above, xenografts may be acquired from long-established cell lines. The dilemma, however, is that such models are excessively reliant on cell lines that have inadvertently been physiologically and biochemically altered through extended growth on plastic structures, which completely lack a functional immune system and are ectopic in nature (Merlino et al., 2013). As a consequence, the efficacy of cell line xenograft models is highly dependent on cell population selection during xenografting. However, the landscape shifts when an *in vivo* passaging approach is applied. In line with metastatic progression, serial *in vivo* passaging of metastatic variants can preferentially select for highly aggressive subpopulations, which provides important opportunities to investigate overtly metastatic cases and the subsequent application of phytotherapeutic intervention, despite the potential for genetic and phenotypic divergence (Kienzler et al., 2025).

Emphasising the importance of cell selection, newer xenograft strategies are employing patient-derived xenografts (PDX). In these systems, fresh melanoma biopsies from primary tumour sites are directly implanted into highly immunodeficient mice. The benefit of this methodology lies in preserving tumour heterogeneity, genetic stability, drug response, and mutational profile, and in improving translational relevance through careful control of experimental parameters, which typically render long-established cell line xenografts poorly predictive (Tentler et al., 2012). Due to their relevant and significant resemblance to human cancer models, PDXs are commonly preferred in biomarker and drug efficacy studies (Gao et al., 2015). Nonetheless, as with all evaluation models, PDXs are not without constraints. Firstly, it has been shown that following first engraftment, the carefully selected-for human tumour microenvironment is restructured and replaced by murine stromal components, implying that subsequent tumour-stroma interactions will occur in more of a mouse than a human context. In addition, the inherent variation between human and murine ligand-receptor signalling pathways may result in the loss or modification of human-specific molecular interactions (Patton et al., 2021). Secondly, the pertinent role of immunological modulation in melanoma progression may be neglected and overlooked by the fact that, by virtue of xenograft models, immunocompromised hosts are required. This prevents the evaluation of immune-mediated tumour effects related to the immunological cascade that follows malignant processes, as well as the testing of modern immunotherapy regimens (Ny et al., 2020).

#### 4.1.4 Genetically engineered mouse models

Genetically engineered mouse (GEM) models have substantially advanced the understanding of melanoma pathogenesis by capturing the essence of genetic alterations that drive human disease (Walker et al., 2011). It is an uncommon occurrence for mice to spontaneously develop melanoma; therefore, targeting specific oncogenic pathways such as BRAF or NRAS mutation and/or the obliteration of key tumour suppressors including CDKN2A or phosphatase and tensin homolog (PTEN) provides an opportunity to mirror the molecular aberrations observed in human melanoma and to stimulate the induction of tumour development at competent sites (Merlino et al., 2013). In contrast to the immunocompromisation required in xenograft models, GEM models incorporate immunocompetent hosts that preserve the complex interplay between tumours and the immune system. In a botanical context, this permits the investigation of medicinal plants and phytochemicals not only for direct antiproliferative effects but also for immunomodulatory and anti-inflammatory properties. Immunocompetent hosts used in GEM models develop melanomas within their native tissue context. This circumvents immunodeficiency sequelae commonly observed in xenograft models (Rebecca et al., 2020). The emergence of immune checkpoint inhibitors in metastatic melanoma management provides further justification for the employment of GEM models due to their capabilities of providing a reputable platform for the analysis of the complex and dynamic interaction between tumour and immune system (Carlino et al., 2021). In addition, by using GEM models, it is now possible to avoid long-standing selection pressures and to enhance unintentional mutational frequency observed during long-term cell culture passaging in *in vitro* models. Further developments in GEM models centre on using mice with uniquely defined genotypes and mutational fingerprints to enhance predictability and reproducibility in studies investigating natural products as alternative interventions for melanoma. These models are capable of providing a controlled *in vivo* system that closely mimics human melanoma, such as the role of intrinsic and extrinsic factors that drive *de novo* tumour initiation, allowing researchers to study tumour development, progression, metastasis and responses to therapies within a fully functional immune environment (Kersten et al., 2017).

#### 4.1.5 Syngeneic models

Serving as another example of a preclinical evaluation methodology that utilises immunocompetent hosts, syngeneic transplantation models combine host immunity with tumour progression in a physiologically relevant setting (Patton et al., 2021). Syngeneic studies, such as the C57BL/6 murine model, involve the transplantation of murine melanoma cells into genetically matched hosts and enable the recognition of the immunomodulatory and antimelanomagenic phytotherapeutic potential of extracts or pure compounds due to host immunocompetency. This system is now widely used as a benchmark platform for evaluating immune checkpoint inhibitors, cytokine-driven treatments, and T-cell-based therapeutic approaches. When melanoma cell type is considered for such investigations, the B16 melanoma series, particularly the highly metastatic B16F10 variant, has demonstrated remarkable efficiency and reliability as a syngeneic platform for testing anticancer responses, metastasis, and therapeutic interventions, given its consistency and voluminous tumour formation in immunocompetent mice and propensity for lung metastasis (Becker et al., 2010). Depending on the experimental objective, tumour cells may be administered subcutaneously to generate a localised solid tumour, intraperitoneally to model disseminated disease, or intravenously to promote metastatic colonisation (Couto et al., 2019).

Unfortunately, as a consequence of the reliance on murine melanoma cells derived from a single inbred strain in the B16 syngeneic melanoma model, there is a noteworthy lack of genetic diversity and mutational complexity in such cells, which is in opposition to the notorious genetic heterogeneity observed in human melanoma systems (Beaumont et al., 2014). It is not surprising that biological and physicochemical differences exist between mice and humans, particularly in cancer biology and in the interpretation of murine versus human models. Variations in pathways and receptors governing growth factor signalling, apoptotic regulation, as well as adhesive molecules have proven to limit the translational relevance of results obtained in murine syngeneic models to human systems (Herlyn and Fukunaga-Kalabis, 2010). Furthermore, due to its aggressiveness and high proliferative activity, the B16 model exhibits rapid growth, which shortens the therapeutic and experimental timeline and limits the potential for chronic assessments. As a result of the lack of development of the chronic inflammatory environment characteristic of human tumours, an inability to conclude on the extent of chronic inflammation and the evolutionary progression of disease over time in such a model remains a fundamental challenge (Sanmamed et al., 2016).

### 4.2 Practical considerations

Medicinal plants and their abundance of phytochemicals have shown considerable promise in treating melanoma; however, translating these natural compounds into tangible therapies is a complex, multifaceted process. Many medicinal plant extracts and phytochemicals show strong *in vitro* effects in cytotoxicity, anti-migratory, and anti-invasive assays; however, these effects do not necessarily translate to humans, thereby limiting their extrapolative potential. To ensure plant-derived candidates with potential anti-melanoma activity are practical, it is important to consider principles such as accurate, effective dosing; bioavailability parameters; consistency between batches and standardisation; and potential adverse effects and toxicity. Using lab models, animal studies, and other preclinical tests helps us understand both their potential and their limits. Raising awareness of these practical details is key to developing safe, effective, and reliable plant-based products for melanoma treatment.

#### 4.2.1 Multi-target advantages

A fundamentally important principle in the sphere of medicinal plant science is the inherent phytochemical complexity present in medicinal plant extracts. In contrast to pure compounds, plant extracts contain diverse populations of specialised combinations of secondary metabolites, such as tannins, flavonoids, terpenoids, and phenolic acids. The potential for synergistic interactions or those of a modulatory nature is immense. These interactions, therefore, possess the potential to enhance bioactivity to a level far beyond what would be predicted from a compound in isolation. Research has confirmed this principle, showing that whole extracts display emergent biological effects that cannot be replicated in a model utilising a pure compound alone. Antagonism, however, is not a foreign term in such spheres and thus interactions may not always produce a more beneficial therapeutic effect. Nonetheless, in addition to the potential additive contribution of phytochemicals in plant extracts, their mere presence and association with one another has shown potential improvements in bioavailability and subsequent efficacy of administered formulations via the alteration of absorption, distribution, metabolism and excretion of co-existing compounds (Ma et al., 2009; Vaou et al., 2022; Wagner and Ulrich-Merzenich, 2009). The broader adaptability of extracts' pharmacological activity is thus an important consideration in multi- versus single-target therapies.

The multifaceted bioactivity profile of plant extracts is particularly relevant in oncology. Metastatic progression has been characterised by a dysregulation in signalling pathways, apoptotic evasion, immunocompromisation, cytokine activation, angiogenesis, as well as enhanced migratory and invasive capabilities (Hołota and Posmyk, 2025). The metastatic cascade is therefore multifaceted and requires a multi-mechanistic therapeutic intervention. As such, the coordinated modulation of several critical malignancy-related pathways simultaneously by means of a phytochemically abundant extract is especially advantageous in aggressive malignancies such as metastatic melanoma (Majrashi et al., 2023).

In addition, numerous combination approaches have demonstrated enhanced anticancer efficacy when extracts were combined with conventional treatments, reinforcing the medicinal potential of multi-component systems (Talib et al., 2022). In the melanoma sphere, combining temozolomide with the water extract of *Solanum nigrum* L. markedly increased cytotoxicity against human A375 melanoma cells to 97.0% ± 3.6%, compared with 31.0% ± 7.5% for temozolomide alone and 55.0% ± 17.6% for the extract alone, indicating a clear synergistic effect (Ling et al., 2016). Although enhanced bioactivity is the intended goal of such combination therapy, there are additional benefits that may not be immediately evident. In addition to synergism, many medicinal plant extracts favour a reduction in resistance capacity, as well as in the frequency and severity of adverse effects, due to protective populations of multi-active phytochemicals. As an example, an aqueous multi-herbal extract (8 plants) used in traditional Chinese medicine was shown to attenuate 5-fluorouracil-induced renal toxicity. The protective effect was associated with a multi-mechanistic reduction in apoptosis and necrosis of renal tubular epithelial cells following chemotherapy. Similarly, a hydroalcoholic extract of *Rubia cordifolia* L. demonstrated nephroprotective activity against cisplatin-induced kidney injury (Hołota and Posmyk, 2025). In addition, the alleviation of resistance to cisplatin therapy has been circumvented with the employment of co-administered natural plant extracts (Dasari et al., 2022). Finally, as a well-known illustration of the multi-mechanistic concept, the European phytotherapeutic formulation Iberogast®, which contains nine whole-plant extracts, has demonstrated therapeutic efficacy equivalent to synthetic conventional agents such as cisapride and metoclopramide. While producing fewer adverse effects and exerting far broader bioactivity, such as modulating gastric motility, influencing gastric acid secretions, autonomic regulation and anti-inflammatory activity, this preparation is unlike single-target synthetic drugs, which typically address only one aspect of the disorder in question (Wagner and Ulrich-Merzenich, 2009). Ultimately, the therapeutic classification of a plant-based extract or formulation as multi-mechanistic depends primarily on the complexity and functional diversity of its phytochemical profile.

#### 4.2.2 Dose and bioavailability challenges

Despite the promising anti-melanoma effects of many phytochemicals *in vitro*, translating these findings into effective therapies requires careful consideration of dosing and bioavailability. Numerous challenges arise, including poor absorption, accelerated metabolism, interactions with metabolic enzymes involved in co-administered drugs, and rapid clearance and low systemic exposure, which reduce the available therapeutic concentration in tumour tissues (Lagoa et al., 2020). As an example of such rapid metabolism and clearance, resveratrol, an infamous bioactive phytochemical, has a half-life of only 10 min, raising the question of how effective its therapeutic effects may be if it is present for such a short period (Jung et al., 2015). Due to the dynamic and extensive phytochemical population present in plant extracts, discrepancies in aqueous solubility and chemical stability have also proven to be a hindrance to achieving optimal dose (Aljabali et al., 2025). Even when a phytochemical is absorbed, many of these unique compounds fail to be efficiently located and accumulated in their intended target tissues. Furthermore, individual differences in pharmacokinetics can further contribute to variability in efficacy. These factors collectively make it difficult to achieve consistent, therapeutically relevant concentrations in the body (Sultana, 2024).

When developing a foundation for the optimisation of a natural product-based therapeutic strategy, a thorough understanding of absorption, distribution, metabolism, and excretion (ADME) is essential in pharmacokinetic research. The lack of detailed pharmacokinetic data for many phytochemicals creates significant challenges in determining appropriate dosing and predicting clinical efficacy (Hochman, 2021). In an attempt to circumvent these challenges, physiologically based pharmacokinetic models are increasingly being developed and integrated into the study of natural product pharmacokinetics to simulate how phytochemicals behave in the human body. This, in essence, supports the development of more effective, targeted formulation strategies (Aljabali et al., 2025).

In addressing and overcoming the challenges associated with the sometimes limited bioavailability of plant extracts and their respective phytochemicals, novel formulation strategies play an integral role, having been shown to improve solubility, enhance stability, and increase tumour-specific and systemic concentrations of these phytochemicals. Examples of such formulation strategies include nanoemulsions, encapsulation and complexation modalities involving structures such as nanoparticles, phytosomes and liposomes (Lagoa et al., 2020). Building on the example of the poor bioavailability of resveratrol, it was found that encapsulating resveratrol in solid lipid nanoparticles significantly improved its solubility, stability, and cellular delivery, thereby enhancing its anti-proliferative effects while preventing cytotoxicity (Teskač and Kristl, 2010). Such approaches also enable more precise dose titration, which is essential for balancing efficacy with safety and ensuring reproducibility across studies.

#### 4.2.3 Extract standardisation and batch variability

One of the foundational challenges in phytotherapy research and development is the lack of an active guarantee of consistent quality and active ingredient composition. Complex mixtures of hundreds of phytochemical compounds, whose concentrations vary substantially, in conjunction with environmental perturbations, soil composition, genotype, season and locality of harvesting and differences in post-harvest processing, all contribute to the noteworthy variability in extract composition, metabolite concentration and subsequent function (Patel, 2020). An example of the influence of characteristics such as differences in plant part, soil type and extraction solvent is seen in the highly varied and heterogeneous concentration of withanolides in *W. somnifera* (Kaundal and Kumar, 2025). These variations are in stark contrast to the consistency and standardisation procedures observed with synthetic drugs, making it difficult to ensure that each batch of extract contains the same profile and concentration of bioactive compounds. Failure to address this irregularity can lead to differences in therapeutic efficacy and safety across batches of natural products. In addition, due to the fact that the phytochemical profile of many plant species is understudied and comprises hundreds of compounds, quality consistency evaluation has proven far more challenging than with synthetic drugs (Xiong et al., 2013).

Standardisation seeks to address these concerns by providing definitions and enforcing consistent levels of selected therapeutically relevant marker compounds. It has been suggested, however, that this process may only be deemed feasible and effective if the entire value chain is investigated, whereby raw material is accurately selected and authenticated, material is stored and processed according to standard operating procedures, formulations are produced in a consistent and safe manner coupled with a final and thorough analytical verification post-production (Kaundal and Kumar, 2025). Without such control, even products labelled as derived from the same plant species may differ substantially in their chemical composition, compromising reproducibility in both preclinical studies and clinical trials. Compounding this challenge is the factor of adulteration and its impact on quality and safety. It has been estimated that between 10% and 80% of medicinal plant products in developing countries have been adulterated (Srirama et al., 2017). Commonly defined as the intentional addition or substitution of a different plant species into a herbal formulation for financial or product volume gain, adulteration has also occurred accidentally as a result of misidentification due to morphological and aromatic similarities and poor cultivation practices (El Beyrouthy and Abi-Rizk, 2013). Botanical products intended for therapeutic use, therefore, require a defined chemical profile to support dosing accuracy, safety assessment, and regulatory approval. In ensuring uniformity, chromatographic fingerprinting techniques such as high-performance liquid chromatography (HPLC), high-performance thin-layer chromatography (HPTLC) and gas chromatography (GC) in conjunction with spectroscopic techniques such as liquid chromatography-mass spectrometry (LC-MS) and nuclear magnetic resonance (NMR) are widely employed to profile phytochemical markers in extracts that will be used for the standardisation process (Noviana et al., 2022; Sharma and Yadav, 2023). These fingerprints enable product manufacturers and researchers to compare raw materials and finished products against reference standards, thereby assisting in botanical identity authentication and consistency monitoring across different production batches. In addition, the inclusion of multivariate analytical schemes has proven highly valuable in such fingerprinting endeavours, while DNA barcoding approaches have also demonstrated their potential for clarifying species identification in efforts to mitigate adulteration (Han et al., 2016; Sheidai et al., 2019; Xiong et al., 2013).

As technology has progressed, it has become clear that traditional standardisation methods have inherent limitations, and that alternative avenues, such as metabolomics-based approaches, are increasingly used to better link the chemical composition of plant extracts to their biological effects. Unlike the conventional chromatographic techniques mentioned above, which focus on specific known marker compounds, metabolite profiling enables the simultaneous analysis of numerous compounds without requiring prior identification of the active constituents (Gupta et al., 2014). Supporting the development of more reliable and biologically consistent extracts, metabolomics represents a systems-level strategy to obtain the most comprehensive chemical fingerprint. Ultimately, establishing reproducible phytochemical profiles across batches facilitates the safe and reliable use of natural products, which have attracted considerable attention in melanoma research of late.

#### 4.2.4 Safety and toxicity

With the advent of medicinal plants as therapeutic interventions for melanoma, the safety profile of these herbal preparations requires rigorous safety evaluation. In addition to standardisation, the toxicological assessment of natural products is often dismissed due to the longstanding use of medicinal plants over millennia, with seemingly no adverse outcome. From a traditional perspective, this is further complicated by the limited availability of long-term toxicity data for the herbal preparations administered by traditional health practitioners and by the misconception that “natural” equals “safe” (Ernst, 1998; Gupta et al., 2014). Compounding this are observations such as an investigation of 1,701 patients admitted to two general medical wards in a Hong Kong hospital, revealing that only 0.2% of admissions were attributed to adverse reactions associated with Chinese herbal medicines, with two cases classified as severe (Chan et al., 1992). Nonetheless, it is well known that toxicity reports from users of natural products remain isolated and infrequent. In light of this uncertainty, comprehensive toxicity testing of plant extracts should include acute, subchronic, and chronic toxicity studies, alongside specialised assays that investigate organ- or system-specific effects, such as genotoxicity, immunotoxicity, reproductive toxicity, and carcinogenic potential. To provide context, Table 4 below summarises the available toxicity profiles of the medicinal plants investigated in this study. The table is designed to facilitate efficient comparison of the toxicity studies conducted for each species by providing the type of toxicity investigated, the toxicity data, the route of administration, and the duration of the experiment. In addition, the specific plant part and type of extract employed in the toxicity study, as well as the plant part and extract utilised in the aforementioned anti-melanoma/anti-metastatic assays, have been documented. This aims to correlate observed bioactivity with the toxicity profile, accounting for inherent phytochemical variation across plant parts and extraction methods. The detailed biological mechanisms and anti-melanoma activities of these plants are examined at each respective stage of the metastatic cascade rather than in Table 4 itself.

**TABLE 4:** An overview of the toxicity profile of 29 medicinal plants with known antimelanoma activity.

Plant name	Toxicity investigated	Toxicity data	Route of administration and experiment duration	Plant part and extract used in toxicity analysis	Plant part and extract used in anti-melanoma studies below	References
*Acacia ferruginea* (DC.) (synonymous with *Senegalia ferruginea* (DC.) Pedley)	Acute toxicity	No signs of toxicity up to 2,000 mg/kg in Wistar albino rats	• Oral • 14 days	• Bark • Hydroalcoholic extract	• Fresh aerial parts • Methanol extract	(Faujdar et al., 2018; Sakthivel and Guruvayoorappan, 2018)
*Acacia nilotica* (L.) Willd. ex Delile (synonymous with *Vachellia nilotica* (L.) P.J.H.Hurter and Mabb.)	Acute and sub-acute toxicity	No acute toxicity in Swiss albino mice. Doses above 250 mg/kg for >28 days may induce hepatotoxicity in Wistar rats	• Oral • Acute = 14 days, sub-acute = 28 days	• Root • Aqueous extract	• Pods • Methanol and chloroform extracts	(Alli et al., 2015; AlQathama et al., 2022)
*Aloe ferox* Mill	Acute toxicity	No acute toxicity with an LD_50_ > 5,000 mg/kg	• Oral • 14 days	• Leaves • Resin extract	• Leaves • Gel and whole leaf extracts	(Celestino et al., 2013; Laux et al., 2022)
*Andrographis nallamalayana* J.L. Ellis (synonymous with *Andrographis beddomei* C.B. Clarke)	Cytotoxicity	IC_50_ value > 120 μg/mL on the noncancerous HEK 293 cell line	• *In vitro* cell culture • 24 h	• Leaves • Methanol extract	• Leaves and stems • Methanol extract	(Goel et al., 2021; Purushotham et al., 2016)
*Aronia melanocarpa* (Michx.) Elliott	Drug-herb interaction	Severe toxicity in a patient treated with trabectedin and concurrent *A. melanocarpa* formulation	• Oral • Unknown	• Berries • Commercial preparation	• Berries • Methanol extract	(Diaconeasa et al., 2017; Strippoli et al., 2013)
*Athenaea velutina* (Sendtn.) D'Arcy	Acute and sub-acute toxicity	Concentrations of 250–1,000 mg/mL revealed no signs of toxicity in female Wistar rats	• Oral • Acute = 14 days, sub-acute = 28 days	• Leaves • Ethanol extract	• Leaves and stems • DCM, methanol and distilled water extract	(Almeida et al., 2020; dos Prazeres et al., 2025)
*Buddleja saligna* Willd	Cytotoxicity	IC_50_ of 91.19 ± 0.69 μg/mL against human dermal fibroblast cells and 54.38 ± 8.55 μg/mL against human keratinocyte cells (HaCat)	• *In vitro* administration • 72 h	• Leaves and stems • Ethanol extract	• Leaves and stems • Ethanol extract	(Twilley et al., 2022 ; 2021)
*Bupleurum chinense* DC.	Hepatotoxicity	Hepatotoxicity in prolonged administration of Radix Bupleuri (dried roots of *B. chinense*)	• Oral • Unknown	• Root • Unknown	• Roots • Ethanol extract	(Hu et al., 2016; Teo et al., 2016)
*Caryocar brasiliense* A.St.-Hil	Acute and sub-chronic toxicity	LD_50_ > 2000 mg/kg in acute toxicity study on Wistar rats. Minor haematological abnormalities in sub-chronic study	• Oral • Acute = 14 days, subchronic = 28 days	• Fresh pulp • Oil extract	• Fruit peel • Butanol fraction of hydromethanolic extract	(Silva et al., 2022; Traesel et al., 2016)
*Clinacanthus nutans* (Burm. f.) Lindau	Sub-acute toxicity	300–900 mg/kg demonstrated no signs of toxicity in male Sprague-Dawley rats	• Oral • 14 days	• Leaves • Methanol extract	• Leaves • Aqueous extract	(Fong et al., 2019; P’ng et al., 2013)
*Cotinus coggygria* Scop	Sub-chronic toxicity	Aqueous infusion at 1, 2% and 4% showed no toxicity in male Wistar rats	• Oral • 30 days	• Leaves • Aqueous infusion	• Leaves • Aqueous fraction of ethanol extract	(Antov et al., 2024; Pavlov et al., 2013)
*Daphne gnidium* L	Larvae mortality study	Mortality of fourth-stage *Culex pipiens* larvae reached 84.26% at 100 g/L	• Administration in assay suspension • 15 days	• Leaves • Aqueous extract	• Leaves • Methanol, butanol and chloroform extracts	(Benhissen et al., 2015; Chaabane et al., 2014)
*Drimys winteri* J.R.Forst. and G.Forst	*Caenorhabditis elegans* toxicity model	Survival reduction at the highest concentrations (25–50 mg/mL) only	• Administration in assay suspension • 48 h	• Aerial parts • Essential oil extract	• Bark • Ethyl acetate extract	(Bruna et al., 2022; Russo et al., 2019)
*Euphorbia helioscopia* L	Sub-acute toxicity	600–1,200 mg/kg over 2 weeks showed no toxicity	• Oral • 14 days	• Leaves and stems/latex • Methanol extract -leaves	• Leaves and stems • Ethyl acetate and n-butyl alcohol fractions of ethanol extract	(Dou et al., 2024; Saleem et al., 2014)
*Garcinia hanburyi* Hook.f	Chronic toxicity	Limited extract toxicity data. Gambogic acid, a key phytochemical in this species, was non-toxic at a dose of 60 mg/kg	• Oral • 13 weeks	• Resin	• Isolated gambogic acid	(Qi et al., 2008; Zhao et al., 2008)
*Graptopetalum paraguayense* (N.E.Br.) E. Walther	Acute and sub-acute toxicity	Acute toxicity - LD_50_ > 1,000 mg/kg in Sprague-Dawley rats. No adverse effects in doses up to 1,000 mg/kg in subacute study	• Oral • Acute = 14 days, sub-acute = 28 days	• Leaves • Aqueous extract	• Leaves • 50% ethanol extract	(Chung et al., 2012; Peng et al., 2023)
*Hibiscus* x *rosa-sinensis* L	Acute and sub-acute toxicity	Acute toxicity - LD_50_ > 2000 mg/kg. No adverse events at 400 mg/kg in sub-acute study	• Oral • Acute = 14 days, sub-acute = 14 days	• Leaves • Methanol extract	• Flowers • Aqueous extract	(Goldberg et al., 2017; Nath and Yadav, 2015)
*Hibiscus sabdariffa* L. (synonymous with *Sabdariffa gossypiifolia* (Mill.) M.M.Hanes and R.L.Barrett)	Acute and sub-chronic toxicity	Acute toxicity: LD50 = 8,750 mg/kg in male mice and 7,500 mg/kg in female mice. Significant adverse events in Sprague-Dawley rats in a sub-chronic study	• Oral • Acute = 14 days, sub-chronic = 90 days	• Calyces • Ethanol extract	• Flowers • Methanol extract	(Su et al., 2018; Susilawati et al., 2023)
*Ixeris dentata* (Thunb. ex Thunb.) Nakai (synonymous with *Ixeridium dentatum* (Thunb.) Tzvelev)	Sub-acute toxicity	No renal or hepatic pathology at 100–200 mg/kg	• Oral • 22 days	• Whole plant • Methanol extract	• Whole plant • Methanol extract	(Lee et al., 2016; Shin et al., 2017)
*Lannea coromandelica* (Houtt.) Merr	Acute toxicity	LD_50_ > 3,000 mg/kg in Swiss albino mice	• Oral • 72 h	• Leaves • Ethanol extract	• Bark • Ethanol and aqueous extracts	(Imam and Moniruzzaman, 2014; Nargatti and Wadkar, 2024)
*Moringa oleifera* Lam	Acute toxicity	Toxicity in Sprague-Dawley rats, only demonstrated at doses >3,000 mg/kg	• Oral • 48 h	• Leaves • Aqueous extract	• Leaves • Ethyl acetate extract	(Asare et al., 2012; Luetragoon et al., 2024)
*Ocimum tenuiflorum* L	Acute toxicity	No toxicity at doses up to 5,000 mg/kg in Sprague-Dawley rats	• Oral • 14 days	• Aerial parts • Hydroalcoholic extract	• Leaves • Hexane, DCM, ethyl acetate and methanol extracts	(Moyo et al., 2024; Murugan et al., 2025)
*Physalis peruviana* L	Acute and sub-chronic toxicity	Acute toxicity - LD_50_ > 5,000 mg/kg in Wistar rats. Increased mortality in male rats at 5,000 mg/kg in sub-chronic study	• Oral • Acute = 14 days, sub-chronic = 90 days	• Fruits • Pressed juice extract	• Fruits • Aqueous extract	(da Silva et al., 2024; Perk et al., 2013)
*Piper longum* L	Acute and sub-acute toxicity	Acute toxicity in Wistar rats - LD_50_ > 2,000 mg/kg. No adverse events in sub-acute testing	• Oral • Acute = 14 days, sub-acute = 28 days	• Fruits • Hydroalcoholic extract	• Fruits • Methanol extract	(Jogdand et al., 2024; Sunila and Kuttan, 2006)
*Prosopis juliflora* (Sw.) DC. (synonymous with *Neltuma juliflora* (Sw.) Raf.)	Acute and sub-acute toxicity	Acute toxicity – LD_50_ = 200 mg/kg. No adverse physiological effects in sub-acute study	• Oral • Acute = 72 h, sub-acute = 30 days	• Stems • Ethanol extract	• Leaves • Methanol extract	(Choudhari et al., 2025; Prabha et al., 2012)
*Sarracenia purpurea* L	Limited data available	Limited data available	Limited data available	Limited data available	• Stems • Acetone extract	(Liu et al.2022)
*Swertia chirayita* (Roxb.) H.Karst	Acute toxicity	In male KM mice, doses up to 2,000 mg/kg displayed no signs of toxicity or mortality	• Oral • 72 h	• Whole plant • Ethanol extract	• Stems • Ethanol extract	(Chen et al., 2011; Thakur et al., 2024)
*Taraxacum officinale* F.H. Wigg (synonymous with *Taraxacum* sect. *Taraxacum* F.H.Wigg.)	Brine shrimp lethality assay	LD_50_ values of 7.12, 10.32 and 14.12 ppm for the methanol, acetone and hexane extracts, respectively	• Administration in assay suspension • 24 h	• Whole plant • Methanol, acetone and hexane extracts	• Whole plant • Methanol extract	(Akhtar et al., 2022; Jeon et al., 2017)
*Zanthoxylum bungeanum* Maxim	Cytotoxicity	Concentration of 0.45% v/v demonstrated an approximate 50% reduction in HaCat cell viability	• *In vitro* administration • 48 h	• Seeds • Pressed oil	• Seeds • Pressed oil	(Pang et al., 2019)

Acute toxicity studies involve administering a single dose of a test substance and monitoring animals for 14 days to assess mortality and determine parameters such as the LD_50_ or the maximum tolerated dose. In contrast, sub-acute and chronic toxicity studies require repeated daily dosing over extended periods, 28–90+ days, to establish safety thresholds such as the no observed effect level (NOEL) and the lowest observed adverse effect level (LOAEL) (Jităreanu et al., 2023). A user-friendly, unified framework for toxicity testing has been established by the Organisation for Economic Co-operation and Development (OECD). The value of these evaluations in the broader landscape of phytotherapy lies in their ability to clarify dose-limiting toxicities and potential long-term risks that may not be clinically apparent during short-term use.

In the context of oncology, such as metastatic melanoma, this is particularly critical to assess due to the recent surge in plant-derived products administered concurrently with cytotoxic chemotherapy, targeted therapy or immunotherapy, increasing the risk of drug-herb interactions and cumulative toxicity (Choudhury et al., 2023). Pharmacokinetic metabolic interactions can influence treatment outcomes in two opposing ways. One pathway involves a reduction in drug clearance following the inhibition of drug-metabolising enzymes, leading to elevated systemic drug concentrations and an increased risk of toxicity. The opposing pathway describes how enzyme induction can accelerate drug metabolism, thereby lowering drug exposure and potentially compromising therapeutic efficacy. Beyond these pharmacokinetic effects, herbal medicines may also interact through pharmacodynamic mechanisms, producing synergistic, additive, antagonistic, or neutral effects that further influence clinical outcomes (Fasinu et al., 2012). An example of an inhibitory metabolic effect on cytochrome P450 (CYP450) enzymes was displayed in a study investigating the safety of *Salvia aurea* L. and *Sphedamnocarpus pruriens* (A.Juss.) Szyszył., where it was determined that CYP2C9 enzyme activity was reduced by 82.75% using the ethanolic extract of *S. aurea* at 50 μg/mL, and at 100 μg/mL, the ethanolic extract of *S. pruriens* reduced CYP2C19 enzyme activity by 75.11% (Seaman et al., 2026). The noteworthy degree of enzyme inhibition in this study has far-reaching implications for herbal preparations, should any such preparations be metabolised by the same CYP enzymes as their conventional co-administered counterparts.

Another form of drug-herb interaction, which is commonly overlooked, is direct physicochemical interaction between the substances themselves and not necessarily the direct systemic effect. The sheer complexity and concentration of phytochemicals present within medicinal plant preparations provide an endless source of potential physicochemical interactions. An incompatibility between a drug and a herb can modify the physical properties of each agent, including stability and solubility. The downstream consequence of such an alteration often manifests as altered bioavailability and a clinical profile markedly different from that observed in investigations testing each agent in isolation (Florence and Attwood, 1998). The difficulty in modelling thousands of phytochemicals against highly specific and targeted conventional therapies and predicting drug-herb interactions provides justification for the limited clinical studies assessing this interaction, where such interactions are primarily studied in an *in vitro* setting (Chan et al., 2023). Furthermore, it has been noted that an isolated herbal constituent may exhibit markedly altered stability and bioavailability profiles compared with a single herb containing multiple phytoconstituents. Synergistic drug-herb interactions are fundamentally based on this physicochemical process, in which simultaneous solubility of both agents and the multiplicity of targets they act on generate a response greater than the sum of the individual agents alone (Carmona and Soares Pereira, 2013). It is thus an important consideration in determining whether to administer an isolated phytochemical or an extract comprising hundreds of chemical constituents.

In addition to pharmacokinetic and pharmacodynamic considerations, systemic immunological responses have moved to the forefront of modern melanoma therapy investigations, particularly regarding their relevance to immune checkpoint inhibitor treatment regimens. Immune checkpoint inhibitors are associated with a diverse yet distinct spectrum of immune-related adverse events, with toxicity exhibiting marked variations when different agents and/or different regimens are considered. While promising, combination therapies have, in some cases, been shown to result in a greater degree of toxicity than immune monotherapy; however, in the case of nivolumab, conventional dacarbazine therapy was shown to exhibit a greater degree of toxicity versus the more favourable safety profile of nivolumab (Robert et al., 2011). Regarding immunological drug-herb interactions, systemic access to herbal preparations is commonly achieved via oral administration. As numerous medicinal plants have demonstrated notable immunomodulatory activity, systemic responses arising from the absorption and physiological processing of herbal preparations may concurrently influence the way in which co-administered drugs are perceived systemically by the immune system, processed to generate an immune response and the subsequent dynamic interaction of this response with the mechanism of action of the concomitant immunotherapy (Grice et al., 2024). Depending on the physiological context, herbal preparations have been shown to enhance weakened immune responses, such as those observed in immunodeficiency or malignancy, or suppress excessive immune activity observed in pro-inflammatory states. Alteration of transcriptional signalling networks, regulation of cytokine production, and modulation of both innate and adaptive immune cell activity are pathways through which herbal preparations mediate immune activity (Singh et al., 2026). When considering the narrow immunological window associated with malignancy, it is essential that co-administered herbs, although immunomodulatory, do not stimulate excessive immune activation and unrestrained immune checkpoint regulation. On the other hand, it is equally essential that immunosuppression be limited, as insufficient restoration of immune function reduces the therapeutic efficacy of conventional therapy (Ramos-Casals et al., 2020; Yiu et al., 2026). As several plant extracts discussed in this review exhibit immunomodulatory effects, careful evaluation of their compatibility with immune checkpoint inhibitors is essential in minimising the risk of immune-related toxicity and altered efficacy. This is particularly true, given that immune-related adverse events observed with immunotherapy have delayed onset and prolonged duration, often hindering the identification of causal drug-herb interactions (Ramos-Casals et al., 2020).

Apart from drug-herb interactions, it is imperative to consider the toxicity profile of the individual extract constituents. In line with recognising the extensive phytochemical diversity, the use of medicinal plants in existing therapies requires careful consideration of toxic metabolites that occur in those species. Compounds such as pyrrolizidine alkaloids are known for their hepatotoxic, phototoxic, genotoxic and cytotoxic effects, whereas furan derivatives have shown carcinogenic and hepatotoxic potential. Most well-known–digoxin, the cardiac glycoside from the foxglove plant, is yet another example of the need for a cautious and informed approach to herbal formulation administration, lacking toxicological analysis (Prajapati, 2024). Due to the fact that plant extracts are composed of multiple interacting bioactive constituents, safety assessments should evaluate both the toxicological profiles of individual phytochemicals and the extract as a whole, rather than considering either in isolation (Aljabali et al., 2025). Moving forward, in an attempt to combat the deficit of toxicity data in medicinal plant usage, enhanced herbal pharmacovigilance systems are becoming increasingly necessary to monitor toxicological assessment and encourage adverse drug event reporting.

#### 4.2.5 Regulatory and intellectual property challenges

While the anticancer potential of phytochemicals is undeniable, a major challenge lies in intellectual property (IP) rights and the subsequent ownership structures should a commercial product be developed. In most cases, it is relatively simple to assign IP rights to a particular party; however, due to the fact that medicinal plants are natural resources and the phytochemicals contained within are naturally occurring, granting exclusive ownership is most challenging (Aljabali et al., 2025). To facilitate the advancement of plant-derived candidates with potential anti-melanoma activity and to prevent a reduction in investment due to IP challenges, collaborative models, such as benefit-sharing agreements, are the cornerstone for protecting traditional knowledge, while balancing commercial exploration and development. A recent success story supporting innovation whilst ensuring equal partnerships amongst stakeholders was in the case of Zembrin®, a bioactive South African botanical product containing *Sceletium tortuosum* (L.) N.E.Br. (synonymous with *Mesembryanthemum tortuosum* L.). In respect of traditional customs and in accordance with respectable practices in intellectual property ventures, the manufacturers of Zembrin® sought to recognise and compensate the San people for their traditional knowledge of *S. tortuosum*. This commitment was formalised through a benefit-sharing agreement with the South African San Council, marking Africa’s first prior-informed consent agreement of its kind (HG&H Pharmaceuticals (Pty) Ltd., 2026). Prioritising collaborations such as this is imperative for the ethical and sustainable development of phytotherapeutics. It ensures that the product’s innovative nature is legally protected and that socially and culturally responsible measures have been implemented in dealings with traditional custodians. Nonetheless, IP protection and recognition represent just one obstacle in phytotherapeutic commercialisation. In addition to IP rights, researchers and manufacturers must also navigate complex regulatory requirements at both national and international levels to ensure compliance and market approval.

There is much variation exhibited in the regulation of herbal products internationally. In the United States, they are classified as dietary supplements and regulated by the FDA under the Dietary Supplement Health and Education Act (DSHEA), with requirements such as good manufacturing practices to ensure safety and quality. In Europe, the European Medicines Agency (EMA) oversees herbal medicinal products, granting either Traditional Herbal Registration or full marketing authorisation based on evidence of safety and efficacy and in accordance with the frameworks prescribed by the Committee on Herbal Medicinal Products (HMPC). In India, herbal medicines are governed by the Ministry of AYUSH (Ayurveda, Yoga and Naturopathy, Unani, Siddha, and Homoeopathy) under the Drugs and Cosmetics Act and its associated rules, which regulate their manufacture and sale (Prajapati, 2024). Strong regulatory frameworks enable quality guarantees, ensuring that natural products are safe, effective, stable, and of consistent quality throughout their time in the value chain. Common areas of investigation within regulatory frameworks include quality assurance, safety monitoring, stability profiles, manufacturing practices and standards, and efficacy evaluation, to reduce the frequency of issues such as contamination, adulteration, mislabelled ingredients, or unsafe products reaching consumers (Wang et al., 2023). Such regulatory frameworks promote public health confidence in the role of plant-derived candidates with potential anti-melanoma activity by supporting therapeutic claims through rigorous safety and efficacy evaluations, including post-marketing surveillance and community-based adverse drug effect reporting.

## 5 Discussion

It is an important observation that although melanoma comprises 1% of all skin cancer diagnoses, its high potential for metastatic activation results in an elevated mortality rate of over 90%, thereby significantly emphasising its clinical relevance. Unlike other variants of dermatological malignancy, such as basal cell carcinoma and squamous cell carcinoma, which typically have a relatively lower metastatic potential and are often manageable with localised interventions, melanoma exhibits a high potential for rapid progression and distant metastasis (Fares et al., 2020; Leung et al., 2012; Nguyen, 2004). Following dissemination beyond the primary tumour site, melanoma exhibits an organotrophic phenotype, thereby chauffeuring tumour cells particularly to organs such as the lungs, liver, brain and bones, where treatment becomes significantly more challenging, and survival rates drop significantly (Costache et al., 2014).

Progression from the radial growth phase to the invasive vertical growth phase marks enhanced metastatic potential (Elder, 2016). Accelerating melanoma development, risk factors such as UV radiation, genetic predispositions to dermatological malignancy, characteristic melanoma mutations and immune dysregulation facilitate proliferation and metastasis (Davies et al., 2002; Menzies et al., 2012; Shain and Bastian, 2016). Growth factors like EGF, VEGF and transforming growth factor beta (TGF-β), along with pro-inflammatory cytokines such as IL-6 and TNF-α, play an integral role in producing an environment which is conducive to metastatic progression (Kalluri and Weinberg, 2009; Rossi et al., 2018). These factors create a tumour-supportive microenvironment that enhances cell survival and promotes the initiation of the metastatic cascade.

Metastasis involves significant ECM degradation by MMPs, intravasation via adhesive proteins such as integrins and cadherins, and survival in circulation through protective tumour cell emboli formation (Chiang et al., 2016; Sznurkowska and Aceto, 2022). Finally, colonisation of distant sites occurs when metastatic cells successfully adapt to new microenvironments, reprogramming their metabolism and modulating immune responses to establish secondary tumours.

Standard treatments for metastatic melanoma, including chemotherapy, radiotherapy, targeted therapy and immune checkpoint inhibitors, have all improved patient outcomes to varying extents (Davis et al., 2019; Maverakis et al., 2015). While targeted therapies like BRAF and MEK inhibitors show initial efficacy, resistance develops quickly as tumours activate alternative survival pathways (Trojaniello et al., 2021). High costs further limit accessibility, especially in resource-poor settings (Karunamoorthi et al., 2012).

Although highly complex and dynamic, the metastatic cascade is marked by significant inefficiencies at multiple physiological stages, which paradoxically contribute to the lethality of metastatic melanoma by enabling only the most adaptable cells to survive. Metastasis begins with local proliferation and invasion; however, not all cells that acquire invasive capabilities succeed in reaching blood or lymphatic vessels, highlighting a critical inefficiency early on in the process. Following on from this, migration into the vasculature via intravasation is yet another notably inefficient step. In an intravasation attempt, cells employ integrins, cadherins and other adhesion molecules to breach endothelial barriers; however, only a fraction successfully enter circulation (Agrawal et al., 2022). Once in circulation, the vast majority of CTCs are rapidly eliminated due to haemodynamic forces and clearance through effective immune surveillance strategies. The harsh shear stress experienced in the bloodstream is capable of causing mechanical damage to tumour cells, further diminishing their survival rate (Chiang et al., 2016).

To counteract these hostile conditions, CTCs often aggregate with platelets, leukocytes and stromal cells in an attempt to form emboli, which provide both mechanical protection and immunological shielding (Van Zijl et al., 2011). Despite these protective mechanisms, the vast majority of CTCs fail to extravasate successfully. Additionally, the tissue microenvironment plays a critical role in determining whether extravasated cells can successfully colonise a new site. Colonisation of distant organs requires tumour cells to adapt to foreign microenvironments, which are often vastly different from their original source. This adaptation entails an extensive amount of metabolic reprogramming, immune shielding, and the recruitment of stromal support. Studies indicate that less than 0.01% of CTCs ultimately establish viable metastases, a reflection of the overwhelming inefficiency inherent to the metastatic cascade (Luzzi et al., 1998).

Despite the inefficiencies throughout the metastatic process, the few cells that successfully complete the cascade often acquire extremely aggressive and invasive phenotypes, necessitating targeted therapeutic strategies to reduce melanoma metastasis. Given the limitations of conventional melanoma therapies, medicinal plants and their phytochemicals have gained attention as viable alternatives for combating metastatic melanoma.

Natural product-based preparations, particularly those derived from medicinal plants, represent a promising avenue for addressing these challenges by offering cost-effective, multi-targeted strategies for melanoma management (Dar et al., 2017; Naeem et al., 2022). The benefit of propagating medicinal plants in a natural environment, with the potential for sustained local cultivation, reduces the need for indefinite reliance on financially prohibitive conventional treatments. Cultural norms and the acceptance of nature-based interventions are also a critical consideration in traditional communities, where, in many developing regions, traditional medicine is deeply ingrained in local healthcare practices (Nandal et al., 2025). The widespread use and trust in herbal remedies, rooted in centuries-old incorporation into medical practice, support the proposal to integrate plant-derived products with antimelanoma activity into existing disease frameworks, even though it is fundamentally important to exercise caution when associating natural products with guaranteed safety.

Throughout the data observed in the biological activity of medicinal plants investigated in this study, it is important to consider the fact that although the employment of specific cell models is essential in furthering understanding, there are some inherent limitations regarding the applicability of studies on non-human cell lines and their extrapolative effects on human cell lines and models. Twelve of the 29 medicinal plants explored in this study utilised B16 murine melanoma cells as their representative melanoma cell lines. Murine models have been instrumental in advancing our understanding of melanoma and evaluating potential lead preparations. However, significant biological discrepancies between mice and humans limit their predictive value in clinical settings, making it impossible to completely emulate the complexity of the whole organism and the microenvironment in which tumours develop. It has been highlighted that differences in immune system function, growth factor composition, invasive enzyme composition, adhesive protein composition, tumour microenvironment and genetic makeup can lead to variations in tumour behaviour and treatment responses between species, necessitating the consideration of the intended downstream user on the proposed botanical formulation (Kuzu et al., 2015).

In addition to the extensive employment of murine models, there has been a particularly concerning overreliance on 2D *in vitro* models for bioactivity evaluation. Although 2D systems remain fundamentally important for high-throughput preliminary identification of lead extracts or compounds, their inability to be evaluated according to ADME pharmacokinetics, inability to replicate tumour heterogeneity, and limitations in extrapolating immunomodulatory activities have prompted alternatives. Even though 3D melanoma model systems are more in line with the architectural complexity of tumour systems, they lack a systemic immunological context. Under circumstances in which xenograft and PDX models are employed, the challenge resurfaces, and the lack of an immunocompetent host severely limits the identification of phytotherapeutics with immunomodulatory potential (Ny et al., 2020). At the opposing end of the spectrum, syngeneic and GEM models are highly relevant because they utilise immunocompetent hosts and can be effectively used to evaluate tumour progression, resistance mechanisms and metastasis. Nonetheless, the upscaling of such intricate evaluation models exposes numerous challenges due to the cost and complexity of some of the techniques involved (Beaumont et al., 2014). Ultimately, no single model fully simulates the dynamic and detailed functioning of integrated human biological systems.

As a result, several practical considerations have been raised, including standardisation and dosing strategies for plant extracts, regulatory compliance, and, most importantly, investigations into the safety profiles of these products.

Despite noteworthy activity, the principle that natural products are inherently safe is a widespread misconception, often exaggerated by the belief that formulations of natural origin are free of side effects and harmful substances. This notion has been consistently challenged in scientific literature, which, over time, has generated a wealth of information drawing attention to confirmed toxic potential and harmful adverse reactions. The controversial trust and belief in the absolute safety of natural remedies is partly rooted in the distrust of synthetic pharmaceuticals (Başaran et al., 2022; Haq, 2004). Furthermore, an overestimation of safety has been nurtured by the consistent, century-old use of medicinal plants. As an example, two medicinal plants mentioned in this study, namely *D. gnidium* and *E. helioscopia*, are known to produce toxic berries and latex, respectively. However, due to centuries of traditional use, the toxic potential of these species has been somewhat overlooked in favour of their promising bioactivity across a range of ailments. In addition, another practical consideration is that, due to the poorly regulated nature of natural products, self-medication is significantly more frequent. Unlike conventional pharmaceuticals, which undergo stringent clinical trials and require strict compliance with regulatory procedures, many natural remedies are readily available as over-the-counter supplements with little to no quality control. Notorious for its lack of standardisation, defining key therapeutic doses has become increasingly challenging as phytochemical variation, even among members of the same plant, results in varied efficacy and safety profiles in medicinal plants (Kunle et al., 2012). Factors such as geographic location, extractive solvent, climate, soil conditions, and the time and season of harvesting can lead to significant fluctuations in bioactive compound levels, affecting the consistency and potency of the remedy (He et al., 2023; Kabubii et al., 2023; Soro et al., 2016).

Nevertheless, even after toxicity has been investigated, several limitations have arisen that actively prevent a homogeneous and effective assessment of safety. From the perspective of *in vivo* toxicity analysis, in the context of this review, it has been observed that numerous animal studies investigated employ relatively small sample sizes and short treatment durations, limiting the statistical power and extrapolative potential. Small sample sizes reduce the ability to detect infrequent adverse effects, which may be critical, whereas short-term investigations often fail to identify cumulative or chronic toxicity profiles that would otherwise only become apparent after prolonged exposure. In addition, such animal studies often utilise concentrations far higher than what may be permitted in human studies. All of the above pose translational challenges, necessitating long-term human trials. Unfortunately, none of the medicinal plants or phytochemicals investigated for their antimelanoma activity in this study is currently in human clinical trials for melanoma. This is a profound limitation when further commercial development is considered. In addition, the toxicity studies available for the medicinal plants investigated in this study have shown marked variation in plant parts selected, extraction solvents used, and methodologies employed. As a result, numerous toxicity investigations have employed plant parts and extracts different from those used in the antimelanoma studies. The heterogeneity of this information presents a comparative dilemma, in which different phytochemical compositions translate into markedly different activities. It is a strong possibility that a species may be disregarded as highly toxic on the basis of the investigation of a single part or extraction solvent, whereas antimelanoma activity data may suggest completely otherwise merely as a result of using an extract with a different chemical composition. Not only is there variability in phytochemical composition, but certain solvents also have specific restrictions in human settings. In this review, methanol was frequently observed as an extractive solvent. Although the use of different extracts is essential for preliminary screening, the application of methanol extracts in human patients is limited when compared to generally recognised as safe (GRAS) solvents such as ethanol. Antimelanoma studies using methanol extracts of medicinal plants may therefore require additional investigation to determine differences in activity and toxicity between the solvents employed.

As an example of variation in biological activity with the extractive solvent, one medicinal plant species in this review, namely *L. coromandelica*, displayed superior antiproliferative activity with the ethanolic extract compared with the aqueous extract (Nargatti and Wadkar, 2024). In addition to this, research comparing multiple solvents and extraction techniques across various medicinal plants shows that methanol, ethanol, acetone and water extract different concentrations and types of secondary metabolites, and that the extraction method (maceration vs. decoction) and plant organ employed change both yield and compound profile (Lezoul et al., 2020). The physiological implication is a notable variation in biological activity and/or subsequent toxicity, leading to inter-batch inconsistencies in commercial settings. A relatively underexplored contributor to variation is that of extractive temperature. In the case of *C. nutans*, a study demonstrated a 5% variation in induced cell death in D24 melanoma cells between hot and cold leaf extracts, which were otherwise produced in identical fashion except for temperature (Fong et al., 2019). This observation stands as a testament to the inherent variability of extracted metabolites across extraction methodologies.

Another key issue facing natural product ventures is the potential for contamination or adulteration. The lack of regulatory obligations has enabled a surge in contaminated plant material, with common contaminants including heavy metals and pesticides. In addition, due to the often polyherbal nature of many botanical products, it becomes increasingly simple to substitute species due to a lack of availability of original plant material, inaccurately identify species in complex formulations, as well as the facilitation of adulteration (Srirama et al., 2017). A potential solution to this challenge is the employment of molecular techniques such as DNA barcoding, which may be used in an attempt to develop a more effective standardisation procedure, assisting with accurate species identification and effective pharmacovigilance (de Boer et al., 2015). Not only does this approach facilitate effective identification, but it also contributes to the maintenance and recording of sustainability-based initiatives and species tracking.

The reality of using natural resources is that medicinal plants are at risk of losing sustainability and diversity due to exploitation and destructive harvesting. Destructive harvesting practices, such as uprooting entire plants, overharvesting from wild populations and unsustainable collection methods, such as bark and root harvesting, threaten the survival of many medicinal species (Chen et al., 2016). It is thus imperative that awareness is fostered in the field of sustainable harvesting methods, cultivation programs, and conservation efforts, which serve as important safeguards, ensuring that medicinal plants remain viable resources for future generations.

From the perspective of the developing world, the relevance of medicinal plants extends beyond mere drug discovery. In resource-limited settings, natural products are often widely utilised due to their relative affordability and cultural acceptability. For many communities, especially those in rural or economically disadvantaged areas, conventional pharmaceuticals may be scarce or prohibitively expensive. Consequently, medicinal plants in many cases serve as primary health care resources, often utilised in accordance with traditional knowledge accumulated over generations (WHO, 2013). When plant-derived candidates with potential anti-melanoma activity for metastatic melanoma are viewed through the lens of the developing world, medicinal plants play a crucial role in modern healthcare by providing bioactive compounds for pharmaceutical drug development.

**FIGURE 4:**
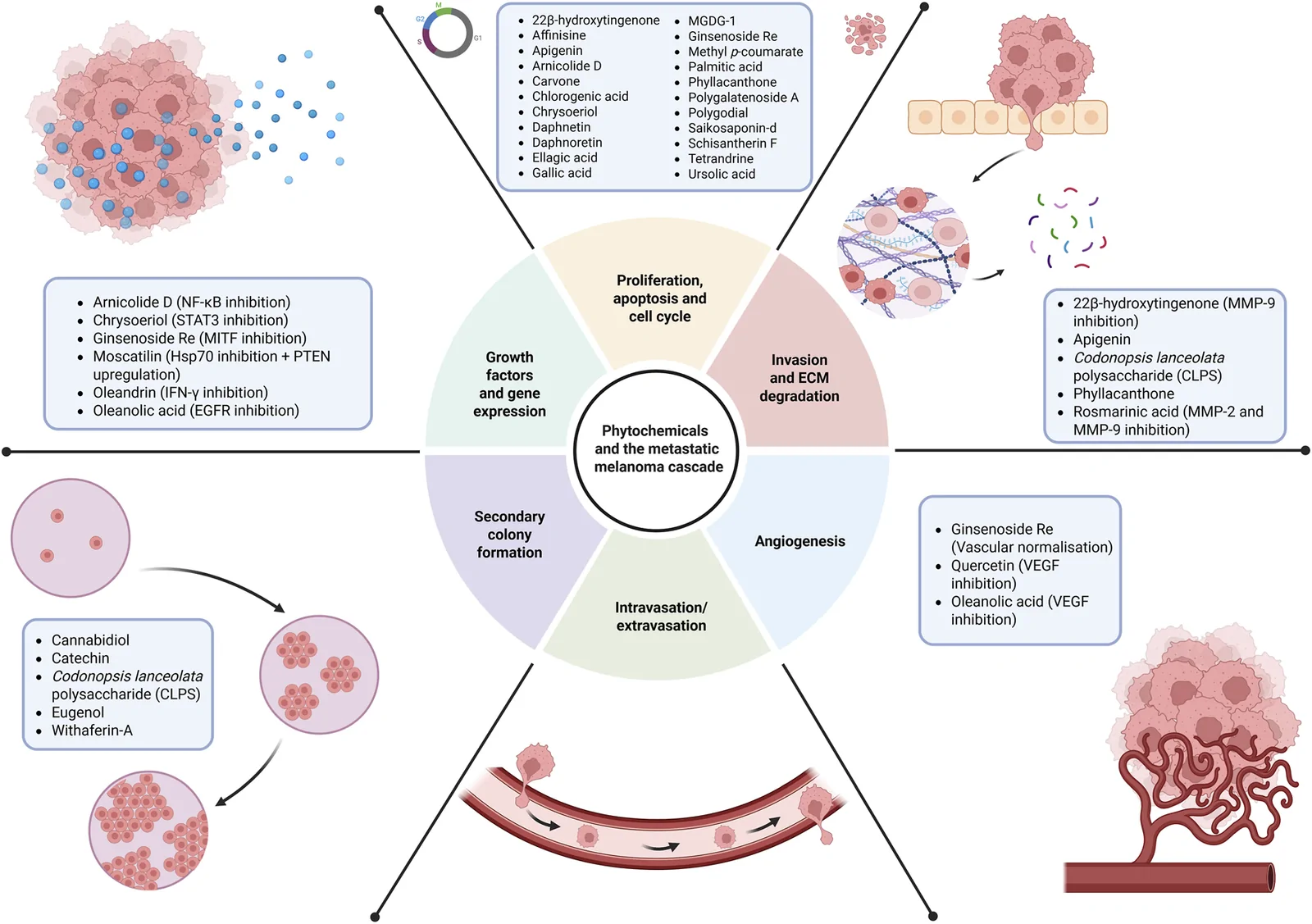
A graphical representation of the antimetastatic capacity of selected phytochemicals at each stage of the metastatic cascade (Created in BioRender. Seaman et al. (2026) https://BioRender.com/161h110).

Considering drug development, when one compares isolated plant compounds with whole-plant extracts, differences in safety and targeting emerge. Purified phytochemicals usually act in a more targeted manner, as researchers are aware of their exact composition and mechanism of action. The downside, though, is that this precision can sometimes come with increased toxicity or side effects. Whole plant extracts, on the other hand, are known to contain a diverse library of compounds, with numerous constituents capable of interacting with one another. In the context of this review, it has been shown that multiple isolated compounds, contrary to their predicted precise targeting, have exhibited multifactorial bioactivity that addresses up to four components of the metastatic cascade. 22β-Hydroxytingenone, arnicolide D, chrysoeriol, oleandrin, phyllacanthone, schisantherin F and tetrandrine have all displayed activity in at least three metastatic sequelae, including components such as apoptotic induction, BRAF inhibition, inhibition of invasive potential, MMP modulation, immunoregulation and cellular signalling modulation (Aranha et al., 2021; de Oliveira-Júnior et al., 2022; Eroğlu Güneş et al., 2022; Gao et al., 2018; Ji et al., 2024; Liu et al., 2023; Ma et al., 2023; Zhu et al., 2019). Even more favourable is that all the above-mentioned compounds were assessed in representative human melanoma cell models. This promotes extrapolative endeavours from human systems to 2D and 3D organ systems, despite the inherent challenges and discrepancies associated with these differences. Despite the multifaceted activities of the compounds listed above, the vast majority of phytochemicals investigated for their anti-metastatic properties in Table 3 exhibited activity only during the proliferative stage of metastasis. Studies addressing invasion, angiogenesis and intravasation were notably limited. Although proliferation is a fundamental initiation stage of metastasis, the metastatic cascade is a prolonged series of events that do not occur in isolation. As a result, the lack of extensive downstream metastatic data, coupled with the absence of melanoma-specific clinical trials, has been identified as a primary and fundamental limitation of this review. In an attempt to highlight the density of antiproliferative studies, the limited available evidence on invasion and angiogenesis, as well as the severe lack of data investigating intravasation/extravasation, Table 3 has been graphically represented in Figure 4.

Although naturally occurring pure compounds are often the preferred choice in the search for novel therapeutics due to their biological origin and are the primary focus of this review, alternative avenues demand further investigation to identify the significant promise that semi-synthetic derivatives hold (Majhi and Das, 2021). The benefits of these semi-synthetic modified compounds are that they can be strategically designed to overcome limitations associated with their natural counterparts, such as poor bioavailability or high toxicity. While this review has maintained a focus on naturally derived phytochemicals with little or no modification, it is important to acknowledge the fact that collaborative ventures aimed at developing universally accessible treatments may require the incorporation of both natural and semi-synthetic approaches (Robles and Romo, 2014).

An example of an emerging and novel angle to drug discovery is that of nanoparticle compositions. Green nanotechnology involves using natural materials, such as plant extracts, fungi or algae, to synthesise nanoparticles. The rationale for employing a green nanotechnology approach is to provide a cost-effective, environmentally friendly means of drug development. These biologically derived nanoparticles are often prized for their lower production costs, enhanced accessibility, reduced off-target effects, and their role as a noteworthy alternative for bridging conventional and traditional medicines (Parveen et al., 2016).

When all of the aforementioned considerations are taken into account, the medicinal plant extracts investigated in this study can be evaluated within a translational framework rather than solely on the basis of *in vitro/in vivo* bioactivity. By implementing this approach, it is possible to identify lead candidates based on the overall strength of evidence for efficacy, toxicity, model type, and clinical progression. Several medicinal plants, such as *A. melanocarpa*, *D. gnidium*, *H. sabdariffa, L. coromandelica* and *P. juliflora* have demonstrated promising anti-melanoma activity in murine cell models, with many exhibiting efficacies across various stages of the metastatic cascade. However, future research on these species should prioritise evaluating these medicinal plants in human melanoma cell lines/models, recognising that a lack of antiproliferative activity, which may be experienced as a result of poor extrapolative potential from murine to human cell models, does not diminish their therapeutic potential, as elucidating underlying metastatic mechanisms remains equally crucial for drug development. Species such as *A. nilotica, A. ferox, B. saligna, B. chinense, I. dentata, O. tenuiflorum, P. peruviana* and *Z. bungeanum* have demonstrated noteworthy efficacy against human melanoma cell lines, enhancing the reliability and applicability of these findings to human populations. A translational roadmap for these lead species highlighting the status of available evidence, as described above, is provided in Table 5.

**TABLE 5:** A summary of the translational status of selected lead medicinal plant species.

Plant species	Extract type	Phytochemistry	Model used in anti-melanoma study	*In* *vivo* melanoma metastasis model data available	Toxicity data available	Melanoma clinical trial data available
*A. nilotica*	Methanol pod extract	Well characterised	*In vitro* cytotoxicity – human cell line	No	*In vivo* acute and sub-acute	No
*A. ferox*	Leaf gel extract	Well characterised	*In vitro* cytotoxicity – human cell line	No	*In vivo* acute	No
*B. saligna*	Ethanol leaf extract	Well characterised	*In vitro* cytotoxicity – human cell line Ex ovo YSM	Yes	*In vitro* only	No
*B. chinense*	Ethanol root extract	Well characterised	*In vitro* cytotoxicity – human cell line	No	*In vivo* chronic	No
*I. dentata*	Methanol whole plant extract	Well characterised	*In vitro* cytotoxicity – human cell line *In* *vivo* murine xenograft	Yes	*In vivo* sub-acute	No
*O. tenuiflorum*	Ethanol leaf extract	Well characterised	*In vitro* cytotoxicity – human cell line	No	*In vivo* acute	No
*P. peruviana*	Aqueous fruit extract	Well characterised	*In vitro* cytotoxicity and wound healing – human cell line	No	*In vivo* acute and sub-acute	No
*Z. bungeanum*	Seed oil extract	Well characterised	*In vitro* cytotoxicity and transwell invasion– human cell line *In* *vivo* murine xenograft	Yes	*In vivo* sub-acute	No

In conclusion, metastatic melanoma remains a formidable therapeutic challenge attributed to its aggressive disease profile, complex metastatic cascade, and capacity to develop resistance to conventional therapies. Although advances in targeted therapy and immune checkpoint inhibitors have notably improved patient outcomes, treatment resistance, adverse effects, and incomplete long-term responses remain fundamental therapeutic challenges. Consequently, there is a pressing need for novel therapeutic strategies which target multiple stages of the metastatic cascade. This has redirected attention to the potential of medicinal plant-based interventions and their secondary metabolites, which exhibit diverse anticancer properties. The medicinal plants and phytochemicals examined in this review have shown considerable promise as novel plant-derived products with antimelanoma activity, exhibiting diverse mechanisms of action that collectively target melanoma cell proliferation, apoptosis, invasion, angiogenesis and metastatic signalling. Unfortunately, the current evidence base is heavily skewed towards a primary focus on *in vitro* studies investigating the stage of proliferation, with comparatively few studies addressing the downstream stages of metastasis, including intravasation, survival in the circulation, extravasation and secondary tumour colonisation. As a result of this imbalance, a major knowledge gap has been identified, and efforts to rectify it should prioritise models that better reflect the biological complexity of metastatic melanoma and integrated physiological systems. Despite encouraging preclinical findings, several important limitations constrain the clinical translation of these strategies. In this study, it was identified that many phytochemicals lack comprehensive pharmacokinetic characterisation, long-term toxicological evaluation, and standardised formulations. As such, generalised assumptions regarding the absolute safety of plant-derived compounds should be avoided, as biological origin does not automatically guarantee a risk-free safety profile. Most notably in this review, despite the breadth of preclinical evidence examined, none of the medicinal plants or isolated phytochemicals discussed have been evaluated in clinical trials for melanoma, representing perhaps the most significant hurdle to clinical interpretation and implementation. It is therefore essential that future research in this sphere prioritises robust *in vivo* studies that effectively emulate the complexity of complete biological systems, the dynamic tumour microenvironment, and the metastatic behaviours associated with it. In addition, thorough pharmacokinetic and toxicological investigations, mechanistic studies of metastasis, formulation optimisation to enhance bioavailability, and representative, comprehensive clinical trials are needed. In order to successfully bridge the gap between promising preclinical evidence and the clinical development of safe and effective medicinal plant-derived therapies for metastatic melanoma, it will be imperative to address these translational barriers jointly.
